# Differential roles for the oxygen sensing enzymes PHD1 and PHD3 in the regulation of neutrophil metabolism and function

**DOI:** 10.12688/wellcomeopenres.19915.2

**Published:** 2024-09-02

**Authors:** Emily Watts, Joseph Willison, Simone Arienti, Pranvera Sadiku, Patricia Coelho, Manuel Sanchez-Garcia, Ailiang Zhang, Fiona Murphy, Rebecca Dickinson, Ananda Mirchandani, Tyler Morrison, Amy Lewis, Wesley Vermaelen, Bart Ghesquiere, Peter Carmeliet, Massimilliano Mazzone, Patrick Maxwell, Christopher Pugh, David Dockrell, Moira Whyte, Sarah Walmsley

**Affiliations:** 1 Institute for Regeneration and Repair, The University of Edinburgh, Edinburgh, Scotland, EH16 4UU, UK; 2 Strathclyde Institute of Pharmacy and Biomedical Sciences, University of Strathclyde, Glasgow, Scotland, G4 0RE, UK; 3 The Bateson Centre, Department of Infection and Immunity and Cardiovascular Disease, The University of Sheffield, Sheffield, England, S10 2TN, UK; 4 Laboratory of Applied Mass Spectrometry, Department of Cellular and Molecular Medicine, KU Leuven, Leuven, Flanders, Belgium; 5 Metabolomics Core Facility, Vlaams Instituut voor Biotechnologie KU Leuven Center for Cancer Biology, Leuven, Flanders, Belgium; 6 Laboratory of Angiogenesis and Vascular Metabolism, Vlaams Instituut voor Biotechnologie KU Leuven Center for Cancer Biology, Leuven, Flanders, Belgium; 7 Laboratory of Tumor Inflammation and Angiogenesis (VIB-KU Leuven), KU Leuven, Leuven, Flanders, Belgium; 8 School of Clinical Medicine, University of Cambridge, Cambridge, England, UK; 9 Nuffield Department of Medicine, University of Oxford, Oxford, England, UK

**Keywords:** Neutrophil, Hypoxia, Prolyl hydroxylase, PHD1, PHD3, Inflammation

## Abstract

**Background:**

Neutrophils are essential in the early innate immune response to pathogens. Harnessing their antimicrobial powers, without driving excessive and damaging inflammatory responses, represents an attractive therapeutic possibility. The neutrophil population is increasingly recognised to be more diverse and malleable than was previously appreciated. Hypoxic signalling pathways are known to regulate important neutrophil behaviours and, as such, are potential therapeutic targets for regulating neutrophil antimicrobial and inflammatory responses.

**Methods:**

We used a combination of *in vivo* and *ex vivo* models, utilising neutrophil and myeloid specific PHD1 or PHD3 deficient mouse lines to investigate the roles of oxygen sensing prolyl hydroxylase enzymes in the regulation of neutrophilic inflammation and immunity. Mass spectrometry and Seahorse metabolic flux assays were used to analyse the role of metabolic shifts in driving the downstream phenotypes.

**Results:**

We found that PHD1 deficiency drives alterations in neutrophil metabolism and recruitment, in an oxygen dependent fashion. Despite this, PHD1 deficiency did not significantly alter *ex vivo* neutrophil phenotypes or *in vivo* outcomes in mouse models of inflammation. Conversely, PHD3 deficiency was found to enhance neutrophil antibacterial properties without excessive inflammatory responses. This was not linked to changes in the abundance of core metabolites but was associated with increased oxygen consumption and increased mitochondrial reactive oxygen species (mROS) production.

**Conclusions:**

PHD3 deficiency drives a favourable neutrophil phenotype in infection and, as such, is an important potential therapeutic target.

## Introduction

In the era of multi-drug resistant organisms, limited development of novel antimicrobials and health threats from emerging pathogens, harnessing our own antimicrobial machinery to better fight infections is an attractive therapeutic option. Neutrophils are essential first responders in the immune system with an arsenal of antimicrobial factors. However, without tight regulation of the neutrophil response, inappropriate or excessive inflammation can lead to significant pathology. Neutrophilic inflammation is implicated in a number of acute and chronic inflammatory disorders, exemplified by acute respiratory distress syndrome (ARDS) ^1^ and chronic obstructive pulmonary disease (COPD) ^2^ in the lung. A more comprehensive understanding of the factors which drive these harmful neutrophil phenotypes, as well as neutrophil antimicrobial capacity, is an essential step in the development of novel therapies for both inflammation and infection.

An important feature of the neutrophil population is their resilience in the face of hostile inflammatory environments. They are highly adapted to not only survive, but to thrive in oxygen and nutrient deplete inflammatory environments. Indeed, hypoxia results in prolonged neutrophil survival ^3^ and enhanced inflammatory responses ^4^.

Cellular adaptations to hypoxia are driven predominantly by the transcription factor hypoxia inducible factor (HIF). HIF activity is regulated by the oxygen sensing prolyl hydroxylase (PHD) enzymes, PHD1, PHD2 and PHD3. In the absence of oxygen, HIFα subunits accumulate, translocate to the nucleus, and form a functional heterodimer with the constitutively expressed HIFβ subunit, leading to transcription of a wide range of genes essential in cellular adaptations to hypoxia ^5^. In the presence of oxygen, the PHDs hydroxylate the α subunit of HIF, targeting it for ubiquitin mediated degradation, thus preventing downstream transcriptional activity. PHDs and HIFα subunits demonstrate significant isoform and context specific activity, affording additional complexity in cellular responses to hypoxia ^6, 7^.

The development of chemical modulators of hypoxic response pathways is a major research focus in this field. A number of pan-hydroxylase inhibitors are either approved for clinical use or currently in clinical trials for treatment of renal anaemia (due to the role of HIF in erythropoietin production) and for inflammatory disorders ^8, 9^. However, the development of truly isoform specific reagents has not yet been achieved. The non-equivalent roles of different HIFα and PHD isoforms is therefore of pathophysiological importance; the outcome of pan-hydroxylase inhibition will depend on the inflammatory context including the dominant isoform and cell type involved.

We have previously shown that PHD2 is the dominant regulator of HIFα in neutrophils under normoxic conditions ^10^. Loss of PHD2 resulted in unchecked HIF activity, enhanced glycolysis and uncontrolled inflammation. Importantly, despite this increased inflammatory capacity, PHD2 deficient neutrophils did not acquire improved bactericidal capacity. In contrast, PHD3 is upregulated in hypoxic neutrophils (consistent with other cell types ^6^) and specifically regulates neutrophil hypoxic survival. Whole animal PHD3 deficiency leads to improved inflammation resolution in hypoxic acute lung injury due to increased neutrophil apoptosis ^11^. The effect of this pro-resolution phenotype on neutrophil antimicrobial responses has not previously been studied and is an important consideration when considering therapeutic targeting of PHD3.

PHD1 has not previously been studied in neutrophils. Knockout of PHD1 has been found to be beneficial in animal models of ischaemic or hypoxic challenge: PHD1 deficiency was protective in models of skeletal muscle ischaemia ^12^, liver ischaemic/reperfusion injury ^13^ and ischaemic stroke ^14^. In each case, the protection was conferred by metabolic alterations, but the specific metabolic adaptations varied with tissue type.

We sought to further delineate the isoform-specific roles of both PHD3 and PHD1 in neutrophils. Hypoxia drives a hyperinflammatory neutrophil phenotype ^15, 16^. Given the evidence from other tissue types that PHD1 deficiency may be protective in hypoxic/ischaemic injuries we investigated whether PHD1 deficiency in the neutrophil population altered inflammation outcomes in hypoxia. We show that, in contrast to other tissues, neutrophil specific PHD1 deficiency is not protective in hypoxic sterile inflammation. Alterations in recruitment dynamics and metabolic flux are seen but are not associated with any identifiable functional differences in this model.

We then investigated the consequences of neutrophil PHD3 deficiency. We expanded our previous findings to include infection models to better understand the global consequences of PHD3 loss. We show that myeloid specific PHD3 deficiency confers enhanced bactericidal capacity in neutrophils with improved *in vivo* outcomes in infection models. Importantly, this is not associated with detrimental hyperinflammatory responses in sterile inflammation. We investigated the potential mechanisms underlying this phenotype and identified an increase in mROS production (with associated increased oxygen consumption in response to bacteria) as a potential driver of enhanced bactericidal capacity.

## Methods

### Generation of mouse lines

Whole animal PHD1 deficient mice and PHD1 floxed mice have been described previously and display no significant health problems ^12^. PHD1 ^fl/fl^ animals were crossed with MRP8-Cre-ires/GFP animals purchased from the Jackson lab to generate neutrophil specific ^17^ PHD1 deficient mice (PHD1 ^fl/fl^MRP8Cre ^+/-^) and Cre negative litter mates (PHD1 ^fl/fl^MRP8Cre ^-/-^). The mice were backcrossed for at least eight generations and maintained on a C57BL/6 background. This line bred normally with no underlying health issues.

Our previous work has utilised whole animal PHD3 deficient mice. We sought to clarify the specific role of neutrophil populations by using myeloid specific and neutrophil specific PHD3 deficient mice. PHD3 floxed mice are previously described ^18^. Myeloid specific deletion was achieved through a lysozyme M-driven (LysM) Cre recombinase ^19^ to generate myeloid specific PHD3 deficient mice (PHD3 ^fl/fl^LysMCre ^+/-^) and Cre negative littermates (PHD3 ^fl/fl^LysMCre ^-/-^), as previously described ^20^. Neutrophil specific PHD3 knockout mice were also used for some *ex vivo* experiments and were generated through crossing PHD3 ^fl/fl^ animals with MRP8-Cre-ires/GFP animals, as above to give neutrophil specific PHD3 deficient mice (PHD3 ^fl/fl^MRP8Cre ^+/-^) and Cre negative litter mates (PHD3 ^fl/fl^MRP8Cre ^-/-^). In both these PHD3 deficient lines, the mice were backcrossed for at least eight generations and maintained on a C57BL/6 background and the mice bred normally with no underlying health issues.

Mice were housed in individually ventilated cages under 12 h light/darkness cycles and controlled temperature (20–23°C) in accordance with UK Home Office guidance. All mice used for experiments were healthy with quarterly and annual testing carried out in accordance with FELASA 2014 Guidelines, using a mixture of environmental, random colony samples and sentinel testing by serology and PCR. Mice had free access to food (Special diets service rat and mouse number 1 maintenance food RMI (P) 801151) and water. For all experiments, both male and female mice were used at age 8–12 weeks. All animal experiments were conducted in accordance with the Home Office Animals (Scientific Procedures) Act of 1986 with local ethics approval.

Blood samples were collected from the inferior vena cava following overdose of intraperitoneal (IP) pentobarbital (250mg/kg) and immediately mixed with 0.5M sterile EDTA at a ratio of 1:9, EDTA:whole blood). For flow cytometry analysis of leucocyte differentials two red cell lysis steps were carried out using RBC lysis buffer (Biolegend) added at a ratio of 1ml per 100μL blood followed by topping up with PBS without magnesium and calcium (Gibco) and centrifugation at 300G for 5 minutes. The resulting cell pellet was resuspended in PBS and cells counted using a BioRad cell counter. They were resuspended at 1x10 ^6^/ml and 200μL aliquots were used for staining. They underwent FC block with TruStain FcX (Biolegend) for 15 minutes on ice then stained with the antibody panel as follows for 30 minutes on ice: Ly6G-Pacific blue (Biolegend 127612), CD3/CD19-PE (Biolegend 100206/152407), SiglecF-PECF594 (BD 562757), CD115-APC (Biolegend 135510), CD45-AF700 (Biolegend 103128). Samples were then washed 3 times with FACS buffer. Appropriate unstained and FMO (fluorescence minus one) controls were run in parallel. Samples were run on a 5 laser LSR Fortessa flow cytometer (Becton Dickinson) and analysed on FlowJo version 10.

### *In vivo* mouse models

***LPS lung injury.*** Mice were treated with lipopolysaccharide (LPS) from *Pseudomonas aeruginosa* (10) (1mg/mL, Sigma Aldrich) for 10 minutes using oxygen driven nebulisation to generate an acute lung injury. Following LPS treatment, animals were housed in standard conditions or were exposed to hypoxia (10% inspired O _2_) in an InVivo Hypoxic Cabinet System (Coy Labs, USA). This sealed system allows animals to be housed in 10% oxygen at room temperature with excess CO _2_ scavenged using Sofnolime soda lime chips (Molecular Products, UK) with colour indicator.

Mice were culled using an overdose of IP pentobarbital (250mg/kg) at 24 or 48 hours post-LPS. Bronchoalveolar lavage (BAL) samples were collected post-mortem through cannulation of the trachea and lavage with 5 aliquots of 0.8mL ice cold 0.9% NaCl.

***Staphylococcus aureus skin abscess model***. Mice were shaved on the right flank and left to recover for 24 hours in standard housing conditions prior to the experiment. On the day of injection, mice were administered 5×10 ^7^ colony forming units (CFU) of SH1000 *Staphylococcus aureus* in 50μL PBS subcutaneously into the right flank. Mice were scored on sickness based on gross external appearance, including assessment of fur ruffling, activity, peri-optic exudate and dehydration.

Mice were culled on day 7 (or at day 2 or 4 for the CFU counts and myeloperoxidase (MPO) measurement) via an overdose of pentobarbital administered intraperitoneally, and the abscess (overlying scab and pustule) excised with a scalpel and weighed. Abscesses were then dissected into three equal pieces, and the pieces weighed and snap frozen and stored for bacterial counts and MPO assay.

***In vivo model of fulminant Streptococcal pneumonia***. Mice were instilled intra-tracheally (IT) with a high dose (10 ^7^ CFU) of *Streptococcus pneumoniae* (type 2, D39) under anaesthesia as previously described ^21^. Following recovery, mice were housed in standard conditions and were culled at 14 hours by overdose of intraperitoneal anaesthetic. Blood samples were collected from the inferior vena cava and BAL was recovered by cannulation of the trachea and lavage with 5 aliquots of 0.8mL ice cold 0.9% NaCl as above.

### Neutrophil isolation

Bone marrow: Both hind limbs were dissected out and stored in a clean bijou container on ice until processed. Each bone was flushed using 10ml of 1xHBSS with 0.2% BSA. The cell suspension was resuspended, passed through a 70μm filter into a fresh 50ml polystyrene falcon tube and then centrifuged at 450G for 10 minutes. 90% vol/vol percoll stock was made using 10X HBSS with NaHCO _3_ and this stock was used to make 81%, 62% and 55% percoll with 1x HBSS. All three percoll preparations were layered in a 15ml falcon tube and the cells were resuspended in 3ml of 1xHBSS with 0.2% BSA which was layered on top of the 52% percoll layer. The gradient was centrifuged at 2000G for 30 minutes (acceleration 1, deceleration 0). Bone marrow monocyte precursors were removed from the top layer with a pastette. Neutrophils were removed from the middle layer and washed in 30ml of 1xHBSS with 0.2%BSA. Hypotonic saline red blood cell lysis was carried out. Following the percoll purification above, neutrophil purity was found to be approximately 90%. In order to generate highly pure bone marrow neutrophils samples, cells from the neutrophil layer were resuspended in 1xHBSS with 0.2% BSA and purified by fluorescence-activated cell sorting (FACS) based on the forward/side scatter profile and auto-fluorescence as previously described ^22^

Bronchoalveolar lavage: Where highly pure BAL neutrophils were required, we used a previously optimized discontinuous percoll gradient coupled with a red cell lysis step in order to gain purities of >98%. BAL cells were first pelleted by centrifugation at 350G for 10 minutes. BAL samples from 1–3 mice (depending on the final cell number required) were pooled onto a single percoll gradient. 90% vol/vol percoll was made using 10X PBS and further percoll solutions were made using this stock 90% percoll mixed with 1X PBS. 3ml of 78% percoll was pipetted into the bottom of a 15ml falcon tube, followed by 3ml of 69% percoll. The cell pellet(s) were resuspended in 3ml of 52% percoll which was then layered onto the 69% layer. The gradient(s) were centrifuged at 1200G for 30mins with an acceleration of 1 and deceleration of 0. Following centrifugation, the macrophages accumulated on the top of the 52% layer and were removed using a sterile pastette. The neutrophils (within the 69% layer) were then removed using a fresh sterile pastette and placed into a 50ml polystyrene falcon tube. This was topped up to 50ml with sterile PBS to wash off the percoll and the purified neutrophils pelleted at 350G for 8 mins. Residual red cells were lysed by hypotonic saline lysis. A cytospin was made prior to the final centrifugation step to assess purity by morphology. During optimisation of this protocol, purity was also confirmed by flow cytometry and the neutrophils were found to be >98% pure.

### Assessing bacterial load/CFU counts

For the *Staphylococcus aureus* skin abscess model, abscess tissue was placed in a sterile dish and cut into small pieces with a sterile scalpel blade. Contents were placed into a sterile bijou container and forceps and scalpel blade rinsed with 1mL PBS into the petri dish. The plate was then washed with a further 1mL PBS and the liquid transferred to the bijou container. The mixture was vortexed for 1 minute and left on ice for 1 hour to allow the scab to dissolve, before vortexing again for 1 minute. 1:10 serial dilutions were made of the homogenate and three 10μL aliquots of each dilution plated onto Columbia Blood Agar plates (VWR). Plates were incubated at 37°C overnight before counting resultant colonies to determine CFUs per lesion and per gram of lesion.

For the *Streptococcus pneumoniae* model, ten-fold serial dilutions were performed on whole blood and BAL aliquots. Three 10μL drops from each of 6 dilutions were then plated onto blood agar plates and cultured overnight in 37°C to calculate viable bacterial counts.

### Assessment of MPO activity in *Staphylococcus aureus* skin abscess model

The abscess was transferred to a sterile screw-cap tube with six homogenisation beads and 0.5mL cold hexadecyltrimethylammonium bromide (HTAB) buffer and the abscess homogenised in a bullet blender for 5 minutes. The homogenate was sonicated for 10 minutes in 30 second bursts then freeze-thawed on dry-ice once to ensure cell lysis. The solution was centrifuged at 4°C and the supernatant transferred to a new sterile tube. 100μL of supernatant was added to 1.9mL of O-dianisidine solution (0.167mg/mL O-dianisidine hydrochloride (Sigma-Aldrich Company Ltd., UK) and the change in absorbance at 450nm from 30 seconds to 90 seconds after addition of supernatant was read on a spectrophotometer (Jenway 6310, Barloworld Scientific, UK) to give relative MPO activity.

### Additional analysis of BAL parameters

BAL cell counts were performed using a haemocytometer and differential counts were assessed by morphology on cytospin preparations. BAL albumin (Abcam Ab108792), IgM (Abcam Ab133047) and elastase concentration (Ab252356) were measured using commercially available ELISA kits. Elastase activity in the PHD1 BAL experiments was measured using the EnzCheck Elastase assay (Invitrogen E12056). MPO activity was analysed using the EnzCheck MPO assay (E33856). BAL inflammatory cytokines were measured using a BD Cytometric Bead Array multiplexed bead-based immunoassay (BD Biosciences): BAL supernatant was collected from BAL following *Streptococcus pneumoniae* infection and analysed for cytokine concentrations by flow cytometry as per manufacturer instructions.

### Bacterial killing assay

Murine neutrophils were isolated from BAL 24 hours following LPS induced lung injury as described above. 1×10 ^6^ neutrophils were resuspended in 200μL glucose free media and plated in a 48-multiwell TC plate. Live SH1000 *Staphylococcus aureus* was opsonised by incubation in 500μL dialysed FBS at 37°C with shaking for 30 minutes. Opsonised SH1000 (multiplicity of infection (MOI) 1:10) was mixed with the neutrophils for a 60-minute co-incubation period. After killing of extracellular bacteria through the addition of gentamycin (2μg/mL) and vancomycin (7μg/mL) for 30 minutes, neutrophils were harvested (T0) or cultured for a further one hour (T1) prior to cell lysis and measurement of internalised bacteria. CFUs were counted and % bacterial killing after 1 hour was calculated.

### Phagocytosis (PHD3 deficient neutrophils)

***CFSE labelling of Streptococcus pneumoniae.*** Aliquots of D39 *Streptococcus pneumoniae* bacteria were centrifuged at 4000G for 5 minutes and then resuspended in 2mL of 10μM Carboxyfluorescein succinimidyl ester (CFSE). The cells were incubated at room temperature for 1 hour in the dark while gently rocking to mix.

After 1 hour, the culture was centrifuged, and the pellet washed three times in PBS and culture adjusted to OD600. Bacteria were heated at 65°C for 10 minutes to heat-kill and stored at 4°C for up to 1 month before use. Heat-killed CFSE labelled bacteria were vortexed to separate clumps. 50μL FBS were added to bacterial suspensions, vortexed and incubated at 37°C for 1 hour to opsonize, then washed twice with 1mL ice-cold PBS. Concentration of bacterial particles was determined through counting in a haemocytometer and concentration of bacteria adjusted to 1×10 ^9^.

***In vitro phagocytosis assay.*** Neutrophils were isolated from murine BAL and resuspended in RPMI +10% FBS at a concentration of 2×10 ^6^/ml. For phagocytosis of D39 *Streptococcus pneumoniae*, 2μL of 1×10 ^9^ CFU/mL bacterial suspension (described above) was then added to cells for an MOI of 10:1. For analysis of staphylococcal phagocytosis, FITC labelled *Staphylococcus aureus* (Wood strain without protein A) Bioparticles were purchased from Invitrogen (S2851) and added at an MOI of 1:1 cells were incubated for a further 30 minutes at 37°C before being aspirated and transferred into 1.5mL Eppendorf tubes, washed in ice-cold PBS once, and then resuspended in ice-cold PBS. Cells were analysed via flow cytometry on the Attune NxT cytometer (Thermo Fisher Scientific).

### Seahorse extracellular flux assays

BAL neutrophils were recovered from PHD3 ^fl/fl^MRP8Cre ^-/-^ and PHD3 ^fl/fl^MRP8Cre ^+/-^ mice 24 hours after LPS induced ALI (as described above). BAL neutrophils were plated in a Seahorse experiment plate which was prepared and equilibrated the day before: A Seahorse XFe24 cartridge was hydrated using 1mL Sodium bicarbonate (0.1M) and stored overnight at 37°C in an incubator without CO _2_. On the day of the experiment, all the wells of an XFe24 assay plate were coated with Cell-Tak (Corning 354240). Neutrophils were left incubating at 37°C for one hour in Seahorse media without glucose and with 2mM L-glutamine (DMEM without phenol red). Heat-killed *Staphylococcus aureus* (SH1000) was opsonised using dialysed FBS and used to infect neutrophils (MOI 1:25) during the assay. The experiment plate was taken out from the incubator 30 minutes prior to the assay and joined with the cartridge plate containing all the compounds for the standard Glycolysis Stress Test which includes the addition of Oligomycin and 2-deoxy-glucose and run with the Seahorse XFe24.

### Neutrophil RNA extraction and TaqMan analysis of gene expression

A minimum of 1×10 ^6^ neutrophils were used to generate RNA samples. The mirVana miRNA isolation kit (Invitrogen) was used to isolate mRNA from highly pure neutrophil samples. The samples then underwent a DNase step using the Invitrogen TURBO DNA free kit. The quantity and purity of the RNA was assessed using a Nanodrop 100 spectrophotometer. cDNA was made using AMV reverse transcriptase and random primers (Promega) with the following settings: 23°C for 5 minutes, 42°C for 2 hours, 99°C for 2 minutes. Gene expression was analysed using predesigned qPCR primer/probe assays and Prime Time Gene Expression Mastermix (IDT, Leuven). For all assays, samples were run in triplicate and the gene of interest expressed relative to expression of a housekeeping gene (β-actin). Assays were run on a 7900HT Fast real-time PCR system (Applied Biosystems) with the following thermal cycling: 50°C for 2 minutes, 95°C for 10 minutes, 40 cycles of 95°C for 15 seconds and 60°C for 1 minutes. Data was analysed using SDS 2.0 software (Thermo Scientific).

Details of primer/probes:

*Egln1* (PHD2): Thermo Fisher Scientific Mm00459770_m1 (exon 3–4)

*Egln2* (PHD1): Thermo Fisher Scientific Mm00519067_m1 (exon 2–3)

*Egln3* (PHD3): Thermo Fisher Scientific Mm00472200_m1 (exon 1–2)

*Actb*: Integrated DNA technologies Mm.PT.39a.22214843.g (exon 5–6)

### HPLC-MS analysis of metabolites

Highly pure BAL neutrophils were analysed for metabolite abundance. Following purification on a discontinuous percoll gradient, cells were washed in ice cold 0.9% NaCl prior to being pelleted (300G for 5 minutes). The pellet was lysed by resuspension in 80% Methanol which had been stored overnight at -80°C and kept on dry ice during resuspension. Prior to analysis, the lysed sample was centrifuged at 20,000G for 10 minutes at 4°C and the supernatant removed into a fresh Eppendorf tube. The pellet was retained, and protein content measured by Pierce BCA assay (Thermo Scientific). Supernatant samples were analysed at the VIB Centre for Cancer Biology in Leuven using a Dionex UltiMate 3000 LC System (Thermo Scientific) coupled to a Q Exactive Orbitrap mass spectrometer (Thermo Scientific) operated in negative mode. Data collection was performed using Xcalibur software (Thermo Scientific). All data values were subsequently corrected for protein content based on the BCA assay of the retained cell pellets.

### Ex-vivo functional assays of PHD1 deficient neutrophils

***Chemotaxis.*** Whole BAL was counted and resuspended at 2×10 ^6^cells/mL. The assay measured movement of cells across a filter towards a chemoattractant (KC, also called CXCL1, for murine neutrophils) using the ChemoTx plate system with 5μm pores (Neuro Probe). The negative control is media with no chemoattractant added. The positive control has cells pipetted into the well at the start (instead of onto the membrane). The percentage of positive control is calculated as (cell count in well for experimental condition/cell count in positive control well)x100. The chemoattractant or control was pipetted into the bottom well of the plate, the filter was then placed on top and the cell suspension pipetted onto the filter. The plate was incubated at 37°C for 1 hour with a lid on. Residual cell suspension was removed from the top of the filter using a cotton bud and the plate centrifuged (without the lid) for 10 minutes at 300G. The filter was removed and the cellular concentration ( *i.e.* the number of cells which have migrated across the filter) was measured in each well using a haemocytometer count of the resuspended well contents. This number was adjusted for the volume in each well to give an absolute cell count.

***Phagocytosis.*** Phagocytosis was assessed by uptake of fluorescently labelled, heat killed *E. Coli* (Invitrogen). BAL neutrophils were resuspended at 1×10 ^6^ cells/mL and incubated at 37°C for 1 hour in RPMI with 10% FCS with the bacteria (MOI 1:1). All samples were washed 3 times with ice cold PBS prior to being resuspended in FACS buffer and analysed by flow cytometry.

***Respiratory burst assay (PHD1 deficient neutrophils).*** *Ex vivo* BAL neutrophils from whole animal PHD1 deficient mice were used for this assay. Total reactive oxygen species (ROS) was measured using a chloromethyl derivative of 2’,7’-Dichlorodihydrofluorescein diacetate (CM-H2DCFDA, Invitrogen). CM-H2DCFDA was resuspended at 50μg/5μL in DMSO. This was then diluted 1:100 with PBS to make up the stock solution. 3μl of this stock solution was added to 100μl of BAL neutrophils at 1×10 ^6^/mL and cells were incubated at 37°C for 45 minutes. To measure respiratory burst capacity, the formylated peptide f-met-leu-phe (fMLF, Sigma Aldrich), was added after 45 minutes for a further 45 minutes at a final concentration of 10μM. The samples were analysed by flow cytometry.

### Additional functional assays in GFP positive PHD3 deficient neutrophils

A number of assays were adapted due to the green fluorescence of the MRP8Cre positive cells in the neutrophil specific knockout mouse lines.

***CellROX assay for total ROS.*** Total ROS in the PHD3 cells was measured using CellROX red rather than the previously described DCF assay. Briefly, BAL neutrophils were isolated 24 hours post-nebulised LPS as previously described. They were resuspended at 1×10 ^6^/mL in glucose free RPMI (consistent with the hypoglycaemic lung microenvironment). CellROX Deep Red Reagent (Invitrogen) was added at a final concentration of 5mM for 30 minutes at 37°C prior to washing and running samples on a 6 laser LSR Fortessa flow cytometer (Becton Dickinson).

***MitoSOX assay for mROS production.*** Cells were isolated and plated as above. MitoSOX red reagent (Invitrogen) was added to samples at a final concentration of 2.5μM for 30 minutes at 37°C prior to washing and running samples on a 6 laser LSR Fortessa flow cytometer (Becton Dickinson).

***SyTox Red assay for NET formation.*** Cells were isolated as above. These activated BAL cells have a high degree of baseline NETosis and NETosis was measured without the addition of a further stimulus. Cells (at a density of 1×10 ^6^/mL) were fixed in 4% PFA for 15 mins at room temperature and then washed in PBS. SyTox Red reagent (Invitrogen) was then added at a final concentration of 0.1mM for 30 minutes at room temperature. Samples were washed again prior to running on the flow cytometer.

All flow cytometry data was analysed using FlowJo V10 and statistical analysis carried out on GraphPad Prism V9.4.

## Results

### Conservation of core neutrophil phenotype and functions in PHD1 deficiency

**Figure 1.**
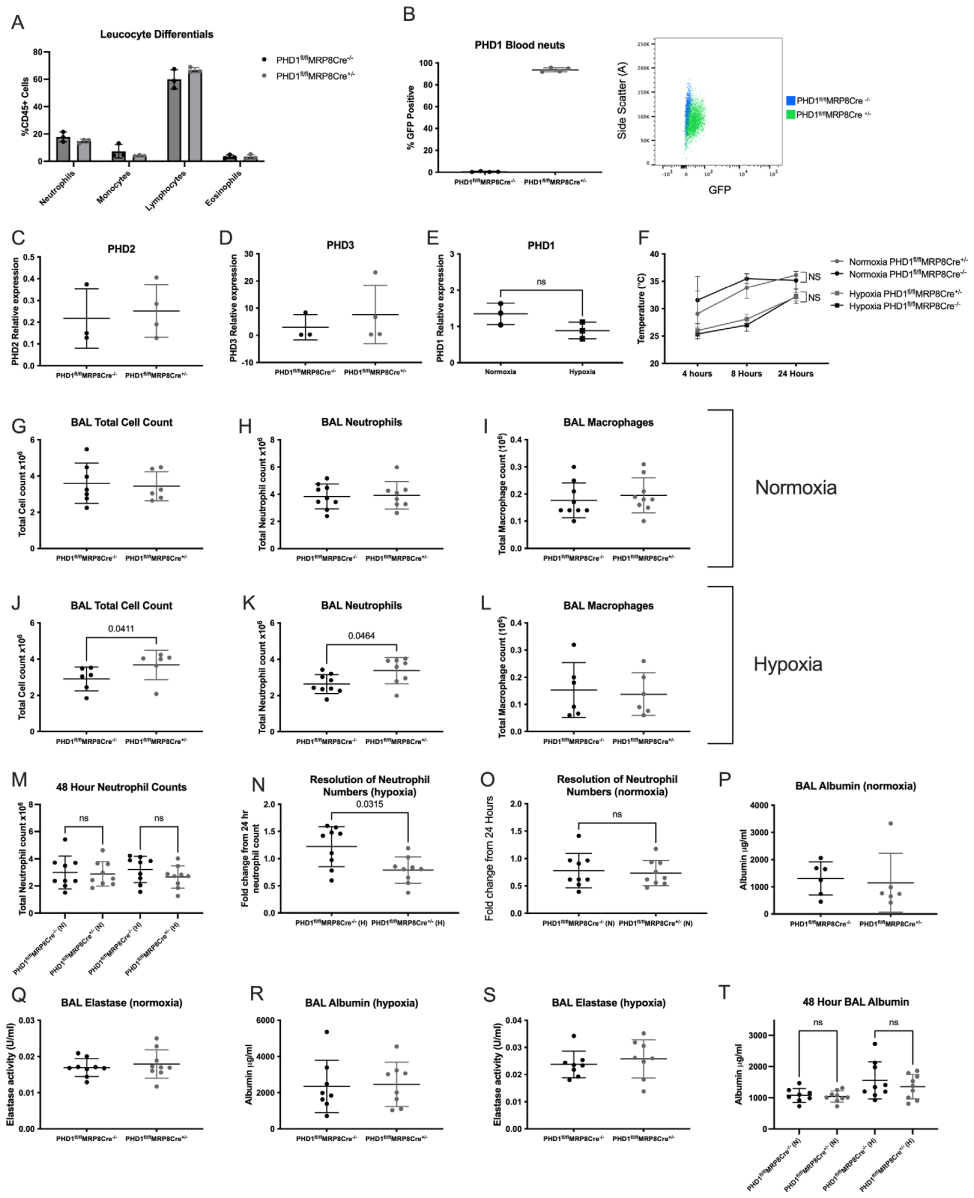
Loss of PHD1 results in oxygen dependent alterations in neutrophil recruitment. Blood leucocyte differentials were determined by flow cytometry ( **A**) and Ly6G positive neutrophils were assessed for GFP positivity ( **B**). Bone marrow neutrophils were isolated from naïve PHD1 ^fl/fl^MRP8Cre ^-/-^ and PHD1 ^fl/fl^MRP8Cre ^+/-^ mice by percoll gradient followed by fluorescence activated cell sorting to ensure sufficient purity. Taqman analysis of PHD2 (egln1) ( **C**) and PHD3 (egln3) ( **D**) gene expression was carried out (N=3 PHD1 ^fl/fl^MRP8Cre ^-/-^, 4 PHD1 ^fl/fl^MRP8Cre ^+/-^). ( **E**) Highly pure BAL neutrophils were isolated from normoxic and hypoxic C57BL/6 wildtype mice 24 hours after LPS induced ALI and taqman analysis of PHD1 (egln2) gene expression was carried out (N=3). ( **C**)–( **E**) expressed as gene of interest relative to β-actin, analysed by unpaired t-test. ( **F**) Core body temperatures of PHD1 ^fl/fl^MRP8Cre ^-/-^ and PHD1 ^fl/fl^MRP8Cre ^+/-^ mice housed in normoxia or hypoxia for 24 hours following LPS induced ALI. N=6, 2-way ANOVA with multiple comparisons (corrected for multiple comparisons by Tukey test). Total BAL cell counts 24 hours after LPS induced ALI of mice housed in normoxia ( **G**) or hypoxia ( **J**), N=6-7. BAL neutrophils counts in ( **H**) normoxia and ( **K**) hypoxia, N=8-9. BAL macrophage counts in **(I**) normoxia (N=9) and ( **L**) hypoxia (N=6). ( **G**)–( **L**) analysed by Mann-Whitney test (unpaired). ( **M**) BAL neutrophil counts were measured 48 hours following LPS induced ALI in PHD1 ^fl/fl^MRP8Cre ^-/-^ and PHD1 ^fl/fl^MRP8Cre ^+/-^ mice housed in normoxia or hypoxia, N=9, ordinary one-way ANOVA with multiple comparisons (corrected for multiple comparisons by Tukey test). ( **N&O**) Resolution of neutrophil infiltration was then calculated from fold change of 48 hour neutrophil counts from the corresponding mean 24 hour neutrophil count, N=9. BAL supernatant collected 24 hours following LPS induced lung injury was analysed by ELISA for albumin ( **P**)&( **R**) and elastase ( **Q**)&( **S**) concentrations (N=6-9). ( **N**)–( **S**) analysed by Mann-Whitney test (unpaired). Albumin concentration was measured by ELISA in BAL supernatant collected 48 hours post LPS induced lung injury in hypoxia **(T**), N=8-9, analysed by ordinary one-way ANOVA with multiple comparisons (corrected for multiple comparisons by Tukey test). All data expressed as mean±SD.

Neutrophil specific PHD1 knockout mice (PHD1 ^fl/fl^MRP8Cre ^+/-^) did not display any overt phenotype at baseline and demonstrated normal circulating leucocyte differentials counts when compared to their Cre negative litter mates ( Figure 1A). A GFP tag is co-expressed with the MRP8Cre and we found that 93.7% of blood neutrophils from PHD1 ^fl/fl^MRP8Cre ^+/-^ were GFP positive compared to 0.44% in PHD1 ^fl/fl^MRP8Cre ^-/-^ controls ( Figure 1B). PHD1 ^fl/fl^MRP8Cre ^+/-^ were compared with their Cre negative litter mates (PHD1 ^fl/fl^MRP8Cre ^-/-^) in an LPS induced acute lung injury (ALI) model. Given the role of PHD1 in other models of hypoxia and ischaemia, and the detrimental effect of hypoxia in the model, we used animals housed in both normoxic (21% inspired oxygen (FiO _2_)) and hypoxic (10% FiO _2_) conditions to replicate the marked systemic hypoxia associated with ALI in patients.

PHD1 deficient bone marrow neutrophils did not show any compensatory upregulation in PHD2 or PHD3 transcripts ( Figure 1C&D). Nor was PHD1 expression increased in hypoxic conditions ( Figure 1E). Following nebulised LPS, animals housed in normoxia displayed mild hypothermia at 4 and 8 hours which fully recovered by 24 hours with no difference noted between genotypes ( Figure 1F). As we have shown previously, concurrent hypoxia was associated with significant hypothermia in this model ^16^ but neutrophil specific PHD1 deficiency did not confer any protection against this ( Figure 1F). In normoxia, PHD1 ^fl/fl^MRP8Cre ^+/-^ animals displayed equivalent bronchoalveolar lavage (BAL) total cell, neutrophil and macrophage numbers ( Figure 1G-H), 24 hours following LPS. In hypoxic animals, PHD1 deficiency was associated with a significant increase in total cell counts at 24 hours ( Figure 1J) which was due to significantly higher neutrophil recruitment ( Figure 1K&L). This difference had resolved by 48 hours where there is no difference between genotypes, irrespective of oxygenation ( Figure 1M). Thus, in hypoxia, PHD1 deficiency was associated with increased but more rapidly resolving neutrophil recruitment ( Figure 1N) with the higher resolution driven by slightly higher 24 hour neutrophils counts in the PHD1 ^fl/fl^MRP8Cre ^+/-^ coupled with slightly lower numbers at 48 hours. This is consistent with our previous work showing that hypoxia results in slightly fewer neutrophils being recruited ^16^, a phenotype which is abrogated in the PHD1 ^fl/fl^MRP8Cre ^+/-^ mice. Resolution in neutrophil numbers was not different between genotypes in normoxic mice ( Figure 1O). Despite this increase in neutrophil recruitment at 24 hours in hypoxia, there was no difference between genotypes in lung damage as measured by BAL albumin leak in either normoxia ( Figure 1P) or hypoxia ( Figure 1R). Neutrophil degranulation was also equivalent with no difference between PHD1 ^fl/fl^MRP8Cre ^-/-^ and PHD1 ^fl/fl^MRP8Cre ^+/-^ in BAL neutrophil elastase in either normoxic or hypoxic conditions ( Figure 1Q&S), despite the higher neutrophil numbers in the PHD1 ^fl/fl^MRP8Cre ^+/-^ mice. Thus, the PHD1 deficient neutrophils may be producing slightly less elastase but there is no net effect of neutrophil specific PHD1 deficiency on either elastase abundance or lung damage (as measured by albumin leak). The more rapid resolution of neutrophil numbers observed in hypoxic PHD1 ^fl/fl^MRP8Cre ^+/-^ mice did not alter lung damage at 48 hours with no difference in albumin leak ( Figure 1T).

**Figure 2.**
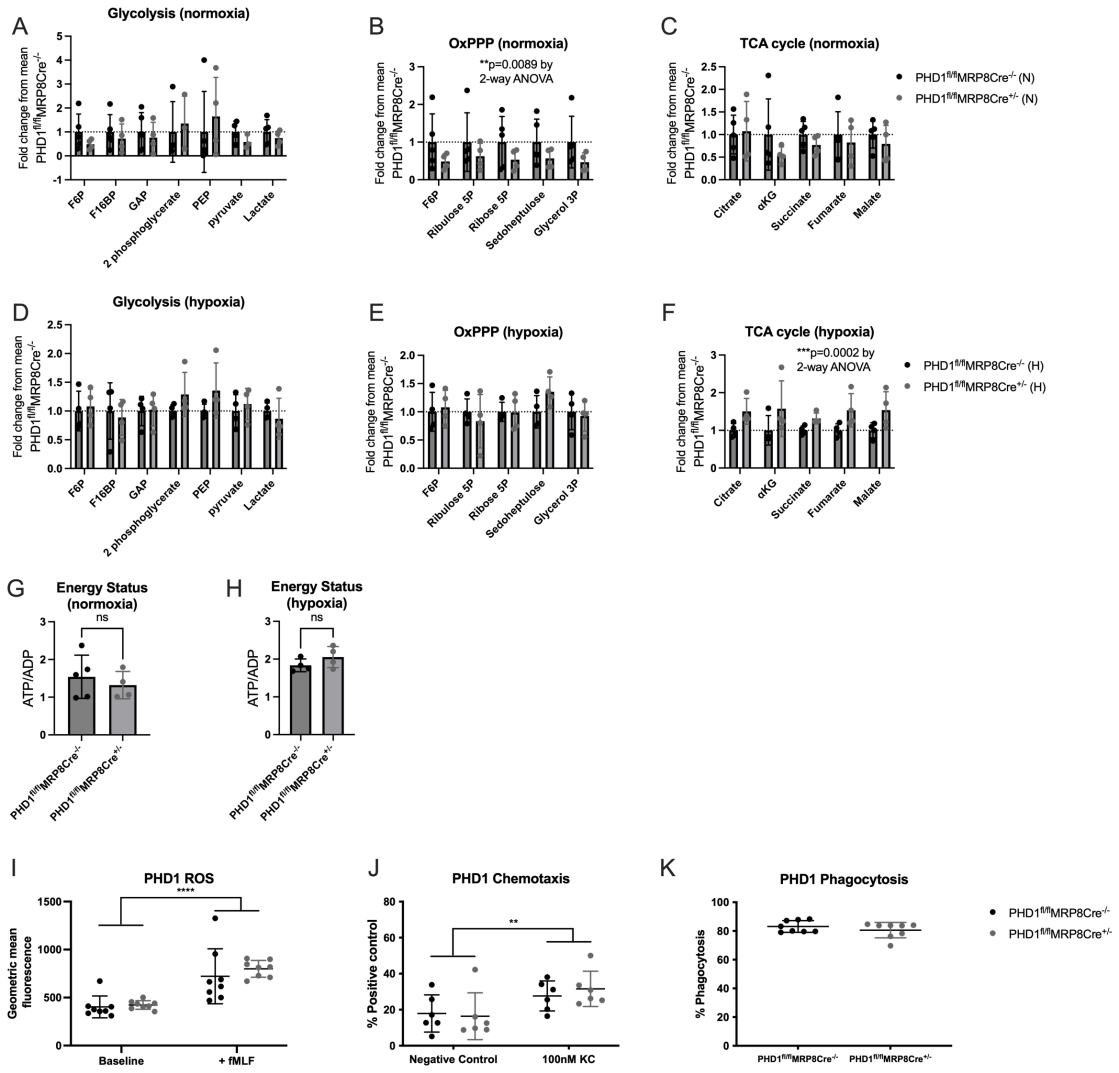
PHD1 deficiency alters metabolic profiles in an oxygen dependent fashion. HPLC-MS analysis of neutrophil metabolic profiles were carried out on *ex vivo* BAL neutrophils harvested 24 hours following LPS induced lung injury in normoxia ( **A**)–( **C**) and hypoxia ( **D**)–( **F**). ( **A**)–( **F**) N=4–5, data expressed as fold change from mean PHD1 ^fl/fl^MRP8Cre ^-/-^ and analysed by 2-way ANOVA. Energy status ( **G**) and ( **H**) expressed as the ratio of ATP:ADP (also measured by HPLC-MS), analysed by Mann-Whitney test (unpaired). *Ex vivo* BAL neutrophils (24 hours post LPS induced ALI) from PHD1 ^fl/fl^MRP8Cre ^-/-^ and PHD1 ^fl/fl^MRP8Cre ^+/-^ mice were analysed for ROS production by flow cytometry following addition of DCF ( **I**), chemotaxis ( **J**) and phagocytosis ( **K**). ( **I**)–( **K**) N=6–8, analysed by 2-way ANOVA. All data expressed as mean±SD. F6P, fructose-6-phosphate; F16BP, fructose-1,6-bisphosphate; GAP, glyceraldehyde 3-phosphate; PEP, phosphoenolpyruvate; αKG, α-ketoglutarate.

We then investigated whether PHD1 deficiency altered neutrophil metabolic profiles. Inflammatory BAL neutrophils were isolated from mice housed in either normoxia or hypoxia 24 hours after LPS induced ALI. In keeping with data from other cell types, PHD1 deficiency did lead to changes in metabolite abundance. Glycolytic metabolites were equivalent between genotypes in both normoxia and hypoxia ( Figure 2A&D). In normoxia, PHD1 ^fl/fl^MRP8Cre ^+/-^ BAL neutrophils had reduced oxidative pentose phosphate pathway (OxPPP) metabolites but preserved TCA cycle metabolites ( Figure 2B&C) whereas in hypoxic neutrophils, OxPPP metabolites were unchanged between genotypes but PHD1 ^fl/fl^MRP8Cre ^+/-^ neutrophils showed significantly higher TCA cycle metabolites ( Figure 2E&F). In post-hoc multiple comparison tests of those pathways showing significant differences, no individual metabolite reached statistical significance, thus there is a small effect across the whole pathway, rather than a significant effect on a select group of metabolites. Despite these changes, energy status (measured by the ATP:ADP ratio from the HPLC-MS) was equivalent between genotypes in both oxygen tensions ( Figure 2G&H), thus increased TCA cycle activity did not lead to an improved energetic profile in hypoxic PHD1 ^fl/fl^MRP8Cre ^+/-^ neutrophils. This is consistent with our previous finding that oxidative phosphorylation contributes minimally to neutrophil ATP stores ^23^. Additionally, despite reduced OxPPP flux in normoxic PHD1 ^fl/fl^MRP8Cre ^+/-^ neutrophils when compared with PHD1 ^fl/fl^MRP8Cre ^-/-^, a corresponding decrease in ROS production was not observed ( Figure 2I). Further analysis of *ex vivo* PHD1 ^fl/fl^MRP8Cre ^+/-^ neutrophils did not identify any significant functional differences when compared with PHD1 ^fl/fl^MRP8Cre ^-/-^ with normal chemotaxis and phagocytosis ( Figure 2J&K).

Thus, despite the important role which PHD1 plays in responding to hypoxic/ischaemic injuries in other tissue types, it does not appear to have a role in the regulation of harmful hypoxic neutrophil responses. Alterations in metabolic profiles do not translate to functional differences. Although the added physiological stress of hypoxia does appear to alter neutrophil recruitment dynamics in PHD1 deficient mice with increased neutrophil recruitment at 24 hours, this is not sufficient to confer any identifiable changes to *in vivo* inflammatory outcomes.

### PHD3 deficiency results in reduced neutrophil recruitment in sterile inflammation

**Figure 3.**
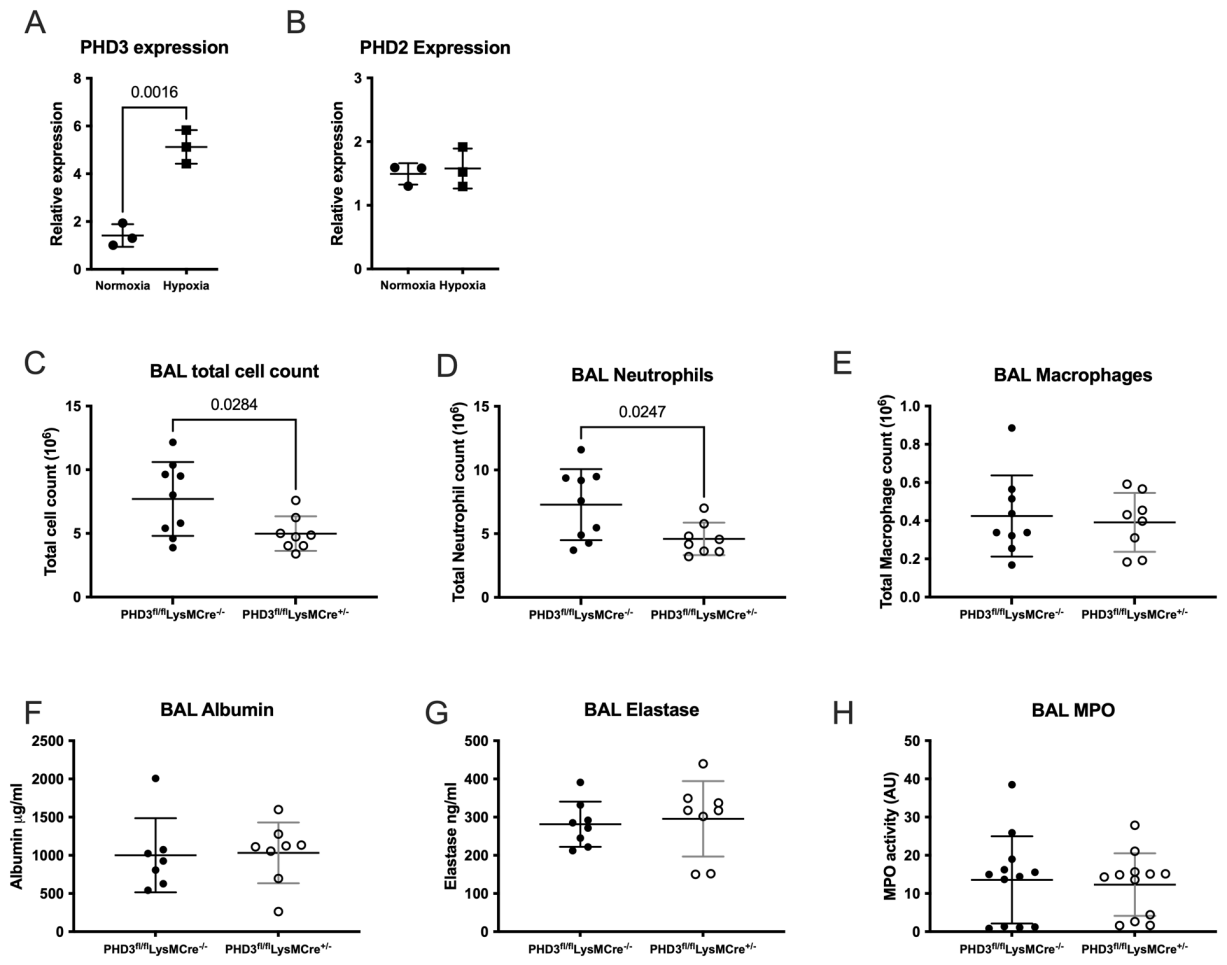
Myeloid specific PHD3 deficiency results in reduced neutrophil recruitment. Highly pure BAL neutrophils were isolated from mice housed in normoxia or hypoxia 24 hours following LPS induced lung injury and taqman analysis of ( **A**) PHD3 ( *egln3*) and ( **B**) PHD2 ( *egln1*) gene expression was carried out. Data expressed relative to β-actin. N=3, unpaired t-test. BAL cells were isolated from PHD3 ^fl/fl^LysMCre ^-/-^ and PHD3 ^fl/fl^LysMCre ^+/-^ mice housed in normoxia 24 hours following nebulised LPS and total cells counts ( **C**), neutrophil counts ( **D**) and macrophage counts ( **E**) were analysed. N=8-9. BAL supernatant was subsequently analysed by ELISA for albumin ( **F**) and elastase ( **G**), N=7-8. MPO activity was analysed by an EnzCheck assay ( **H**), N=12. ( **C**)–( **H**) analysed by Mann-Whitney test (unpaired). All data expressed as mean±SD.

In contrast to PHD1 and PHD2 expression and consistent with previous findings ^6^, PHD3 was significantly upregulated in the BAL neutrophils of hypoxic WT mice following LPS induced lung injury ( Figure 3A&B). We have previously shown that in sterile lung inflammation whole animal PHD3 deletion may be associated with enhanced inflammation resolution in hypoxia ^11^. We have now extended these findings in a LysMCre driven, myeloid specific PHD3 deficient mouse to understand whether PHD3 deficiency alone, without the additional physiological stress of hypoxia, altered inflammation outcomes. In the context of LPS induced ALI in normoxic conditions, PHD3 ^fl/fl^LysMCre ^+/-^ mice showed signs of a suppressed neutrophil response, with fewer total cells recruited to the airways at 24 hours post-LPS ( Figure 3C), due to reduced neutrophil numbers (mean neutrophil number in PHD3 ^fl/fl^LysMCre ^-/-^ 7.26 x10 ^6^ Vs 4.6 x10 ^6^ in PHD3 ^fl/fl^LysMCre ^+/-^ mice, Figure 3D&E). This is in contrast to neutrophil PHD2 deficiency in which we have previously reported an exaggerated and persistent neutrophil response following LPS challenge ^10^ and with neutrophil PHD1 loss, as detailed above. Lung damage, evidenced by albumin leak into the BAL supernatant, was equivalent between genotypes ( Figure 3F), as was degranulation with equivalent BAL supernatant neutrophil elastase ( Figure 3G) and MPO ( Figure 3H).

### Myeloid specific PHD3 deficiency results in improved infection outcomes

**Figure 4.**
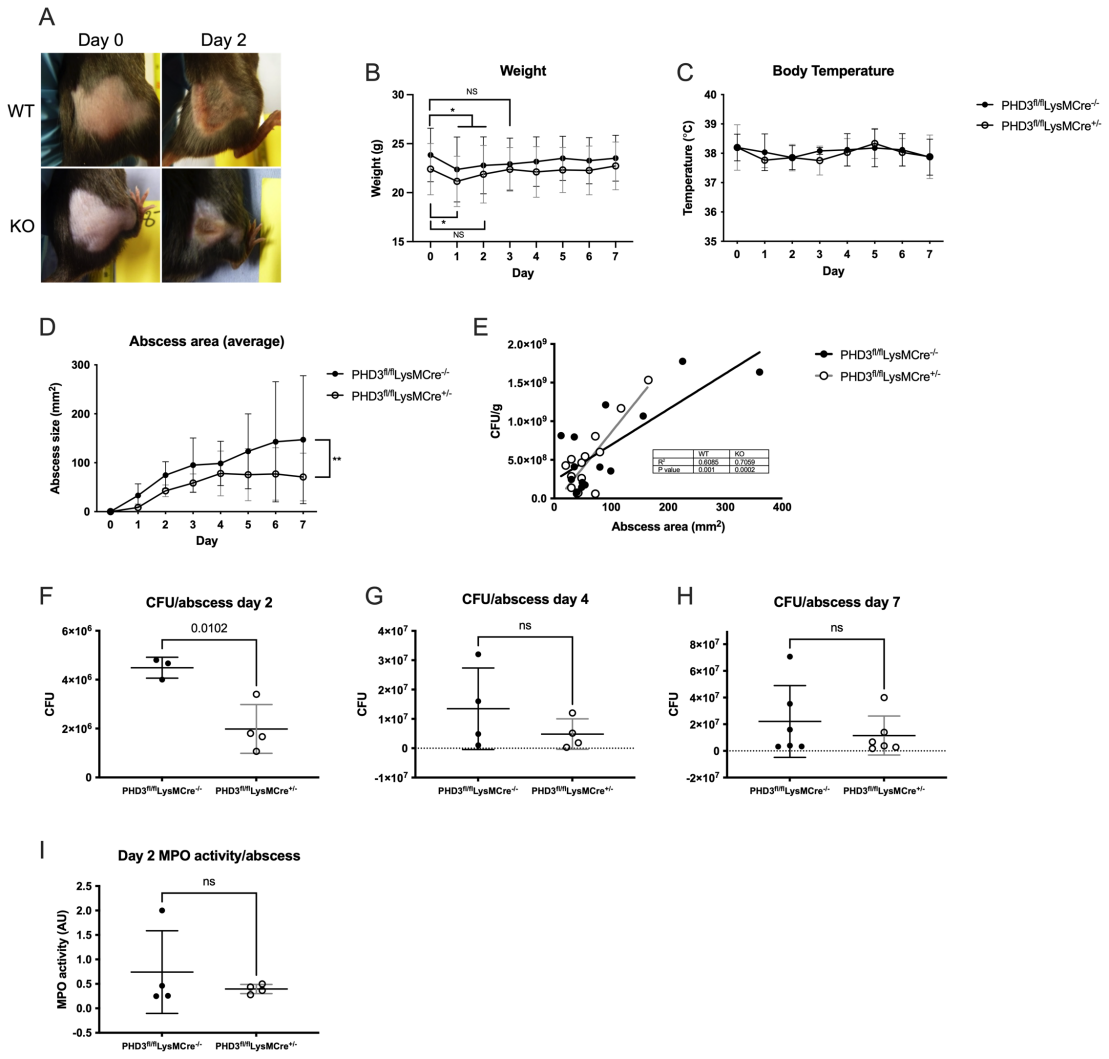
Improved staphylococcal infection outcomes in PHD3 deficiency. PHD3 ^fl/fl^LysMCre ^-/-^ and PHD3 ^fl/fl^LysMCre ^+/-^ Mice were inoculated with *Staphylococcus aureus* subcutaneously into the right flank and abscesses developed over the course of 7 days, with noticeable abscesses by day 2 (typical appearances shown in ( **A**)). Weight ( **B**), rectal temperature ( **C**) and abscess size ( **D**) were measured daily. ( **B**)–( **D**) N=6, analysed by 2-way ANOVA with multiple comparisons (corrected for multiple comparisons by Tukey test). CFU/g and abscess area are significantly correlated (measured by Pearson’s rank coefficient ( **E**), N = 14. Mice were culled at day 2 ( **F**), 4 ( **G**) and 7 ( **H**), abscesses homogenised and viable bacteria counted. N=3 for PHD3 ^fl/fl^LysMCre ^-/-^ day 2 due to exclusion of an outlier (see Figshare data upload for details), N=4 for days 2 and 4 and N=6 on day 7. MPO activity was measured in homogenised abscess tissue ( **I**), N=4. ( **F**)–( **I**) analysed by unpaired t-test. ( **B**)–( **D**) and ( **F**)–( **I**) expressed as mean±SD.

We then investigated how myeloid specific PHD3 deficiency might impact acute responses to infection. We used an established model of *Staphylococcus aureus* skin abscess ^15^ in myeloid specific PHD3 ^fl/fl^LysMCre ^+/-^ mice and their Cre negative littermates (PHD3 ^fl/fl^LysMCre ^-/-^) and measured sickness scores, weight, body temperature and abscess size on days 1–7 to monitor the health of the animals and progression of infection. Neither genotype showed evidence of systemic illness at any point during the experiment (sickness scores consistently zero in both groups, data not shown), even with the development of large abscesses extending to an area greater than 1cm ^2^ (typical abscess appearance shown in Figure 4A). There was no difference in baseline body weight between genotypes. Both genotypes lost a small but significant amount of weight in the first 1–2 days ( Figure 4B) but returned to baseline weight by day 2 (PHD3 ^fl/fl^LysMCre ^+/-^) and day 3 (PHD3 ^fl/fl^LysMCre ^-/-^). Core temperatures were equivalent between genotypes over the course of the 7 days of infection ( Figure 4C). However, in PHD3 ^fl/fl^LysMCre ^+/-^ mice, abscess development was significantly reduced compared to PHD3 ^fl/fl^LysMCre ^-/-^ controls ( Figure 4D). Bacterial burden was assessed by carrying out CFU counts on excised and homogenised abscesses. We found that abscess size was proportionate to bacterial burden in both genotypes ( Figure 4E). PHD3 ^fl/fl^LysMCre ^+/-^ mice had lower total CFU counts at day 2 ( Figure 4F) but this difference was not statistically significance by days 4 and 7 ( Figure 4G&H). Measurement of MPO activity within the abscess did not show a significant difference between genotypes ( Figure 4I), suggesting that the improved bacterial control is not due to enhanced neutrophil degranulation.

**Figure 5.**
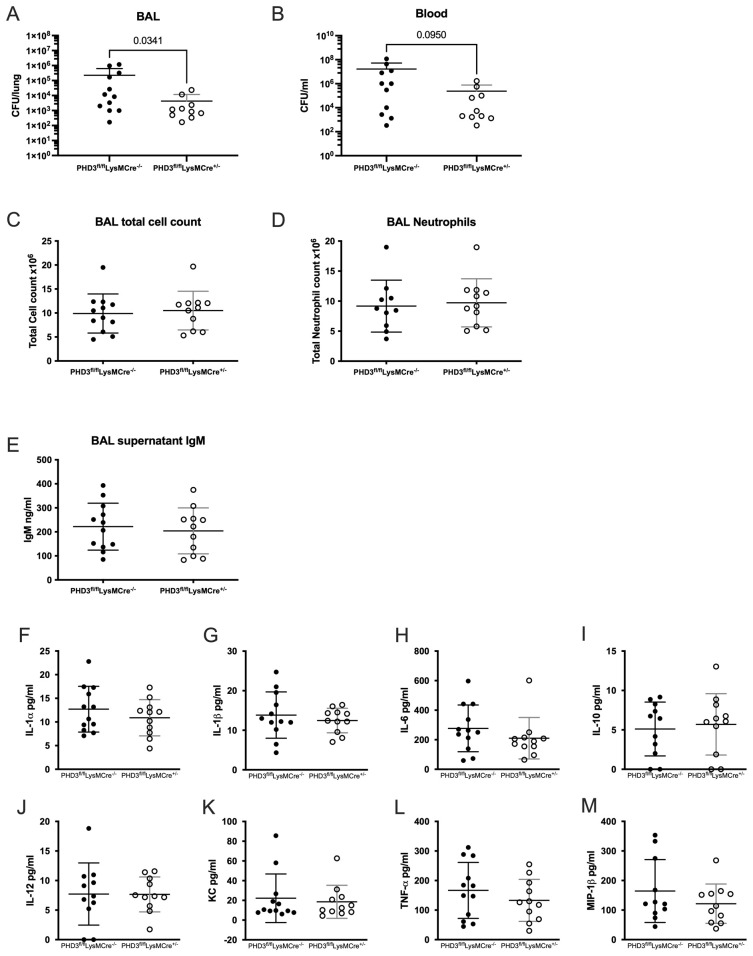
PHD3 deficiency results in reduced bacterial load in pneumococcal infection. PHD3 ^fl/fl^LysMCre ^+/-^ and Cre negative littermates (PHD3 ^fl/fl^LysMCre ^-/-^) were inoculated with *Streptococcus pneumoniae* intratracheally and culled after 14 hours. Bacteria were grown from BAL ( **A**) and blood ( **B**) samples, and BAL cell counts ( **C**) and neutrophil differentials ( **D**) calculated. BAL supernatant was analysed by ELISA for IgM concentration ( **E**). N=10-12, all analysed by Mann-Whitney test (unpaired). ( **F**)–( **M**) BAL supernatant from PHD3 ^fl/fl^LysMCre ^+/-^ and PHD3 ^fl/fl^LysMCre ^-/-^ mice subjected to IT *Streptococcal pneumoniae* infection was isolated after 14 hours and analysed by cytokine bead array for abundance of chemokines and cytokines. N=13-14, analysed by Mann-Whitney test (unpaired). ( **A**) and ( **B**) downward error bars not shown due to logarithmic scale. All data expressed as mean±SD.

We next expanded our findings to include a *Streptococcus pneumoniae* model. Again, PHD3 ^fl/fl^LysMCre ^+/-^mice demonstrated reduced local bacterial burden with significantly lower CFU counts in the BAL in comparison to PHD3 ^fl/fl^LysMCre ^-/-^ controls, 14 hours following intra-tracheal (IT) inoculation ( Figure 5A) although there was not a statistically significant reduction in blood CFU counts ( Figure 5B). This enhanced local bacterial control was not associated with increased total cell ( Figure 5C) or neutrophil ( Figure 5D) recruitment to the airways. Nor was it associated with increased lung damage (as measured by airway IgM leak ( Figure 5E) or increased concentrations of inflammatory mediators in the airways ( Figure 5F–M), suggesting that enhanced bacterial control is not achieved at the expense of increased tissue damage.

PHD3 loss in the neutrophil compartment, therefore, confers a potentially advantageous phenotype with reduced local bacterial burden without excessive tissue damage or persistent sterile inflammation.

### Improved bacterial killing is associated with increased oxygen consumption and mROS production in PHD3 deficient neutrophils

**Figure 6.**
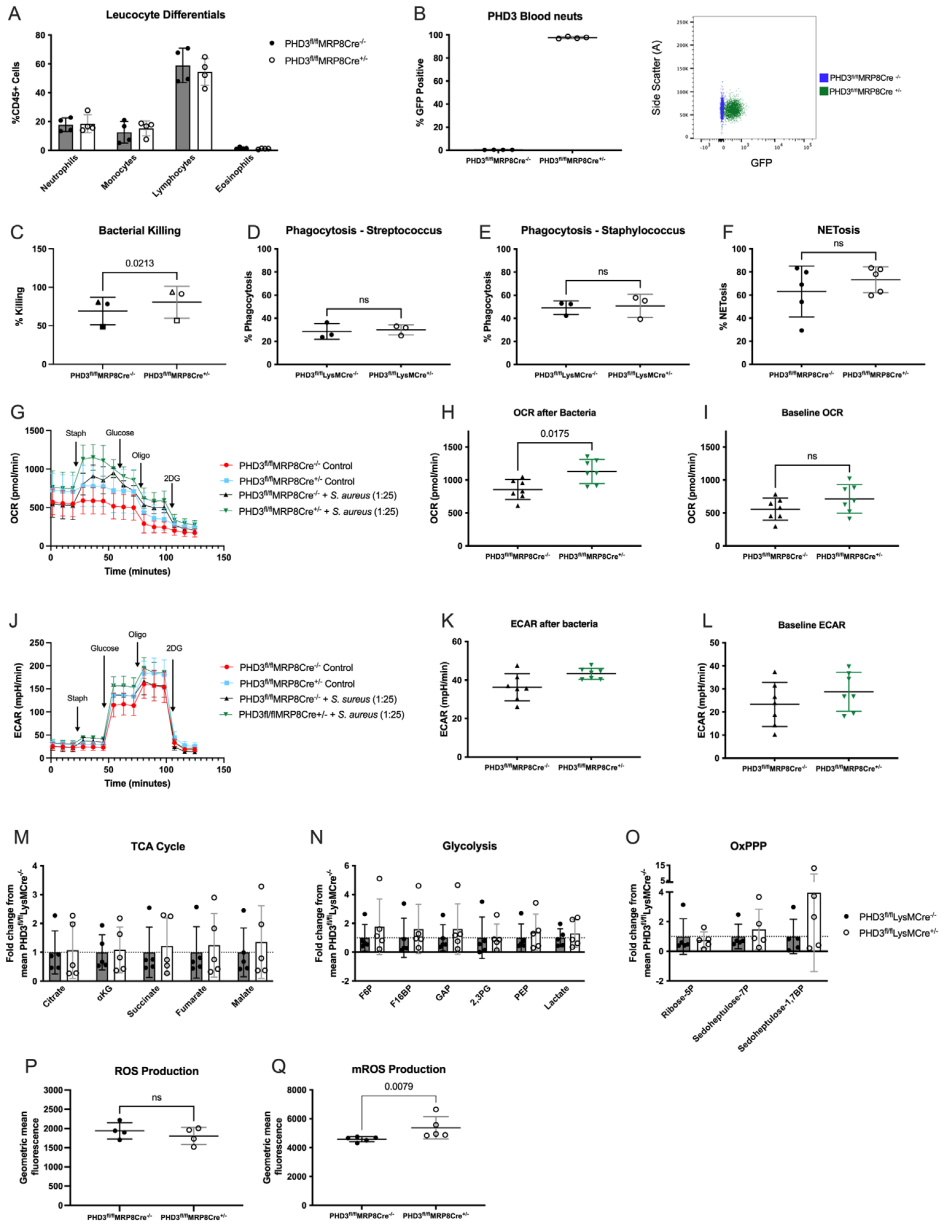
PHD3 deficient neutrophils demonstrate increased oxygen consumption and mROS production. Blood leucocyte differentials were determined by flow cytometry ( **A**) and Ly6G positive neutrophils were assessed for GFP positivity ( **B**). *Ex vivo* BAL neutrophils were isolated from PHD3 ^fl/fl^MRP8Cre ^+/-^ and PHD3 ^fl/fl^MRP8Cre ^-/-^ ( **C**, **F**, **G**- **L**, **P**- **Q**) or PHD3 ^fl/fl^LysMCre ^-/-^ and PHD3 ^fl/fl^LysMCre ^+/-^ mice ( **D**- **E** & **M**- **O**) 24 hours following LPS induced ALI. ( **A**) Bacterial killing was measured using *ex vivo* BAL neutrophils (24 hours post-LPS induced ALI) cultured with opsonised *Staphylococcus aureus* bacteria. Cells were either lysed following washing (T0) or after a further one-hour incubation (T1) and % killing calculated. N=3 over 3 separate experiments, paired t-test (with each experiment designated by a different symbol). Phagocytosis of fluorescently labelled Streptococcus ( **D**) and Staphylococcus ( **E**) was measured by flow cytometry. N=3, analysed by unpaired t-test. ( **F**) NETosis was measured using SytoxRed reagent and analysed by flow cytometry (N=5), analysed by Mann-Whitney test (unpaired). Metabolic flux was measured using Seahorse technology. OCR ( **G**- **I**) and ECAR ( **J**- **L**) were analysed before and after the addition of heat killed *Staphylococcus aureus*. N=7 per condition, **H**- **I**& **K**- **L** analysed by Mann-Whitney test (unpaired). ( **M**)–( **O**) HPLC-MS analysis of neutrophil metabolic profiles were carried out on *ex vivo* BAL neutrophils harvested 24 hours following LPS induced lung injury. N=5, data expressed as fold change from mean PHD3 ^fl/fl^LysMCre ^-/-^ and analysed by 2-way ANOVA. ( **P**) ROS production was measured by incubation of *ex vivo* BAL cells with CellRox red and measured by flow cytometry, data expressed as geometric mean fluorescence. N=4. ( **Q**) mROS production was measured by incubating *ex vivo* BAL neutrophils with mitoSOX reagent and analysed by flow cytometry, data expressed as geometric mean fluorescence, N=5. ( **P**)&( **Q**) analysed by Mann-Whitney test (unpaired). All data expressed as mean±SD. F6P, fructose-6-phosphate; F16BP, fructose-1,6-bisphosphate; GAP, glyceraldehyde 3-phosphate; 2,3PG, 2,3 biphosphoglycerate; PEP, phosphoenolpyruvate; αKG, α-ketoglutarate.

In addition to the myeloid specific PHD3 knock out mice described above, we have developed an MRP8Cre driven neutrophil specific knock out mouse line. We found that circulating neutrophil and other leucocyte numbers were normal in naïve mice in this line ( Figure 6A) and that neutrophils effectively expressed the MRP8 driven Cre, as measured by the GFP positivity (as above for the PHD1 MRP8 Cre mice) ( Figure 6B). To delineate the mechanisms by which PHD3 deficient neutrophils promote antimicrobial control, we cultured *ex vivo* BAL cells from the neutrophil specific PHD3 ^fl/fl^MRP8Cre ^+/-^ mouse lines with *Staphylococcus aureus*. We used glucose deplete media to culture these airway cells to represent the hypoglycaemic airway ^16, 24, 25^. We confirmed a significant increase in bacterial killing by PHD3 ^fl/fl^MRP8Cre ^+/-^ neutrophils ( Figure 6C). *In vitro* we observe this enhanced bacterial killing to be independent of phagocytosis ( Figure 6D&E) and NETosis ( Figure 6F)). However, we did observe a more marked increase in local oxygen consumption rate (OCR), measured by Seahorse, in response to bacteria ( Figure 6G–I)). This uplift in OCR was observed in the context of conserved ECAR (a surrogate for glycolysis, Figure 6J–L), and equivalent abundance of TCA cycle, glycolysis and OxPPP metabolic intermediaries, as measured by HPLC-MS ( Figure 6M–O). As in Figure 2A–F, we also performed post-hoc multiple comparison tests and found that no individual metabolite was significantly different between genotypes. We therefore considered whether this increase in oxygen consumption rates reflected increased ROS production. Total ROS production was equivalent between genotypes ( Figure 6P) but mROS production was significantly higher in PHD3 deficient neutrophils in comparison to Cre negative cells ( Figure 6Q).

Mitochondrial ROS (mROS) are produced by complexes I and III of the electron transport chain and are an important effector of antimicrobial responses ^26^. They have been shown to be induced by infection and to contribute to cytokine production in response to infection in macrophages ^27, 28^. Thus, an increase in mROS production represents a potential mechanism whereby PHD3 deficient neutrophils achieve enhanced bacterial killing associated with increased oxygen consumption upon exposure to bacteria.

## Discussion

Cellular adaptations to hypoxia are critical in maintaining homeostasis and responding to pathogenic environments. Complexity within this system is conferred through the presence of multiple isoforms of the key HIF and PHD proteins which in turn respond differently in different cell types and environments ^7^. We investigated the role of PHD1 and PHD3 in neutrophil responses, having previously demonstrated the critical role which PHD2 plays in neutrophilic inflammation ^10^.

In other cell types PHD1 has been shown to be important in responding to hypoxic and ischaemic insults ^12, 13^. These responses are mediated by metabolic rewiring in PHD1 deplete cells, although the precise changes are cell type dependent. We have shown that PHD1 deficient neutrophils also demonstrate metabolic changes with increased OxPPP metabolites in normoxia and increased TCA cycle metabolites in hypoxia. Further work will be required to elucidate reasons for these differences. In other cell types, the mechanisms underlying PHD1 dependent metabolic shifts include alterations in HIF-1α target expression such as pyruvate dehydrogenase kinase 1 (PDK1) in skeletal muscle ^12^ and increased expression of TP53-inducible glycolysis and apoptosis regulator (TIGAR) in neurons ^14^. We have not further dissected the mechanisms in PHD1 deficient neutrophils but such cell type dependent metabolic rewiring will be of interest in the future. In a hypoxic acute lung injury model, neutrophil specific PHD1 deficiency was associated with increased neutrophil recruitment at 24 hours but more rapid resolution, restoring equivalent neutrophil numbers by 48 hours post-challenge. Alterations in neutrophil metabolic profiles did not lead to changes in inflammatory outcomes, irrespective of oxygenation. Thus, unlike skeletal myocytes and hepatocytes, neutrophil specific PHD1 deficiency does not confer protection against the hyperinflammatory phenotype associated with hypoxia in acute lung injury. Further work is required to interrogate the mechanisms which underly the metabolic shifts observed in PHD1 deficient neutrophils and whether there are any downstream functional consequences. In light of the findings described in PHD3 deficient neutrophils, it will also be of interest to investigate whether neutrophil PHD1 deficiency alters outcomes in infection models.

The protective phenotype in PHD1 deficient muscle fibres and hepatocytes relates to reduced oxygen consumption and consequent reduced oxidative stress. Unlike these highly aerobic cells, neutrophils utilise glycolysis to generate ATP at baseline as well as in hypoxic environments ^21^. Additionally, the generation of ROS, via the NADPH oxidase associated with the phagolysosome, is critical to the neutrophil’s antimicrobial response ^29^. Neutrophils are likely to have different mechanisms of protection against oxidative stress, with compartmentalisation of ROS and effective redox buffering. These fundamental differences in metabolic phenotype may explain why neutrophil PHD1 deficiency does not confer the same protection against a hypoxic insult as is observed in other cell types.

PHD3 deficiency did have a significant impact on inflammation outcomes. Myeloid specific PHD3 deficiency led to a potentially highly advantageous phenotype with improved outcomes in infection without any evidence of excessive sterile inflammation. Using a neutrophil specific mouse line, we further examined PHD3 deficient neutrophils *ex vivo*. We showed a small but significant improvement in bacterial killing in the PHD3 ^fl/fl^MRP8Cre ^+/-^ neutrophils, consistent with *in vivo* findings in the myeloid specific mice. This was not related to increases in NETosis or total ROS production. Using Seahorse technology, we identified enhanced oxygen consumption in PHD3 deficient neutrophils upon challenge with bacteria which we have demonstrated is associated with increased mROS production in inflammatory BAL neutrophils. We therefore suggest that loss of PHD3 confers a beneficial neutrophil phenotype with enhanced mROS production resulting in improved bacterial control, without any identifiable hyperinflammatory phenotype in the context of sterile inflammation. It will be critical to confirm that the *ex vivo* enhanced bacterial killing observed in the neutrophil specific mouse model does translate to improved *in vivo* outcomes, as were seen in the myeloid specific mouse line. In light of this important phenotype, future work should also include investigation of the interface between mitochondrial metabolism, hypoxic signalling pathways and host antimicrobial capacity. It will also be of interest to assess how PHD3 deficiency impacts the activity of different HIF isoforms. These data suggest that PHD3 represents a potential therapeutic target. In the era of multi-drug resistant infections, strategies which target host responses to regulate pathogen control are of huge importance. Fine tuning the immune response via the closely linked hypoxic response system is one potential route to this. In the absence of isoform specific pharmacological PHD inhibitors, the impact of pan hydroxylase inhibition will be dependent on the dominant cell types involved and context of any inflammatory or infective response. It will be important in future work to confirm the role of PHD3 in human, as well as mouse neutrophils. This may be addressed using human neutrophil like cells lines which are amenable to genetic manipulation but ultimately will require specific inhibitors of PHD3. An important future challenge therefore is the need to achieve isoform specific targeting.

## Limitations

Our Study identifies an important neutrophil phenotype but we acknowledge there are some limitations.

We have used both neutrophil specific and myeloid specific mouse lines in these studies and this does limit our capacity to directly compare PHD3 and PHD1 deficiency in neutrophil populations. Our aim in this paper was not necessarily to directly compare PHD1 with PHD3 deficiency, rather to present the data from both genotypes for interest and completeness. Our in vitro data using the PHD3 ^fl/fl^MRP8Cre line is consistent with our *in vivo* data which was carried out in the LysMCre line however, a priority for future studies will be to ensure that the *in vivo* phenotypes do hold true in a neutrophil specific mouse model. Whilst the MRP8 model is ideal to identify the specific impact of neutrophil PHD3 loss, the use of the myeloid specific LysMCre line is also of value. When considering the effect of pharmacological inhibition of PHD proteins, the overall effect on inflammation outcomes will be determined by the sum effect on all infiltrating cells, including monocyte/macrophage cells. The *in vivo* findings of enhanced bacterial control in the LysMCre mouse line are consistent with the *ex vivo* increased bactericidal capacity (caried out on MRP8Cre, neutrophil specific knock out cells) therefore demonstrating that targeting PHD3 *in vivo* may have a net beneficial effect. In order to strengthen these findings further, we have included both lung and skin infection models to ensure the phenotype is broadly relevant. We also acknowledge that we do not have parallel unfloxed but Cre ^+/-^ controls for the mice described here. This would have required us to breed and maintain additional mouse lines. We have previously utilised both LysM ^10, 30^ and MRP8 ^23, 31^ driven Cre and do not identify a Cre specific phenotype. We therefore determined that using these additional controls was not justified when working within the 3Rs framework ^32^.

At present our findings demonstrate an association between bactericidal capacity and enhanced mROS production. Further work will be required to establish the causative role of mROS in the *in vivo* and *ex vivo* phenotypes we have described here. We do not see significant changes in neutrophil recruitment in the pneumonia model in PHD3 ^fl/fl^LysMCre ^+/-^ mice but have not been able to exclude this in the skin abscess model. In future work, intravital imaging may provide a route to measure neutrophil numbers and dynamics in this setting. Additionally, although the loss of PHD1 does not appear to alter neutrophil behaviour, it will be important to confirm this in infection models in addition to the LPS induced ALI model described here and to investigate other potential consequences of the metabolic shifts which are observed.

## Data Availability

Figshare: Differential roles for the oxygen sensing enzymes PHD1 and PHD3 in the regulation of neutrophil metabolism and function, https://doi.org/10.6084/m9.figshare.24106686.v4 ^33^. This project contains the following underlying data: PHD1 Normoxia mass spec raw data PHD1 Hypoxia mass spec raw data PHD1 Normoxia mass spec corrected for BCA data PHD1 Hypoxia mass spec corrected for BCA data PHD3 mass spec raw data PHD3 mass spec corrected for BCA data Fig1A–Fig6Q Data underlying corresponding figures Figshare: ARRIVE checklist for ‘ *Differential roles for the oxygen sensing enzymes PHD1 and PHD3 in the regulation of neutrophil metabolism and function*’, https://doi.org/10.6084/m9.figshare.24106686.v4 ^33^. Data are available under the terms of the Creative Commons Attribution 4.0 International license (CC-BY 4.0).
