# Supplementary material for: Differential roles for the oxygen sensing enzymes PHD1 and PHD3 in the regulation of neutrophil metabolism and function
Source: Wellcome Open Res. 2024 Sep 2;8:569. Originally published 2023 Dec 11. [Version 2] doi: 10.12688/wellcomeopenres.19915.2 (PMC11384204; doi:10.12688/wellcomeopenres.19915.2)
Supplement: Peer review history

# Reviewer report: Reviewer response for version 2

**DOI:** 10.21956/wellcomeopenres.25257.r96741

**Published:** 2024-10-09

**Authors:** Martin Pelletier

**Affiliations:** 1 CHU of Québec Research Center, Quebec, Canada

The authors have adequately addressed my previous concerns.

Is the work clearly and accurately presented and does it cite the current literature?

Yes

If applicable, is the statistical analysis and its interpretation appropriate?

Partly

Are all the source data underlying the results available to ensure full reproducibility?

Yes

Is the study design appropriate and is the work technically sound?

Partly

Are the conclusions drawn adequately supported by the results?

Partly

Are sufficient details of methods and analysis provided to allow replication by others?

Partly

Reviewer Expertise:

Neutrophils, immunometabolism, inflammation

I confirm that I have read this submission and believe that I have an appropriate level of expertise to confirm that it is of an acceptable scientific standard.

# Reviewer report: Reviewer response for version 2

**DOI:** 10.21956/wellcomeopenres.25257.r96744

**Published:** 2024-09-10

**Authors:** Eileen Uribe-Querol

**Affiliations:** 1 Universidad Nacional Autónoma de México, Mexico City, Mexico

I confirm that I have reviewed this submission and, find it suitable for indexing.

Is the work clearly and accurately presented and does it cite the current literature?

Partly

If applicable, is the statistical analysis and its interpretation appropriate?

Partly

Are all the source data underlying the results available to ensure full reproducibility?

Partly

Is the study design appropriate and is the work technically sound?

Partly

Are the conclusions drawn adequately supported by the results?

No

Are sufficient details of methods and analysis provided to allow replication by others?

No

Reviewer Expertise:

Neutrophil functions, developmental biology

I confirm that I have read this submission and believe that I have an appropriate level of expertise to confirm that it is of an acceptable scientific standard.

# Reviewer report: Reviewer response for version 2

**DOI:** 10.21956/wellcomeopenres.25257.r96742

**Published:** 2024-09-09

**Authors:** Frederik Denorme

**Affiliations:** 1 Emergency Medicine, Washington University in St Louis School of Medicine, St. Louis, Missouri, USA

I have no further comments.

Is the work clearly and accurately presented and does it cite the current literature?

Yes

If applicable, is the statistical analysis and its interpretation appropriate?

Yes

Are all the source data underlying the results available to ensure full reproducibility?

Yes

Is the study design appropriate and is the work technically sound?

Yes

Are the conclusions drawn adequately supported by the results?

Partly

Are sufficient details of methods and analysis provided to allow replication by others?

Yes

Reviewer Expertise:

Neutrophil biology in the context of (sterile) inflammation.

I confirm that I have read this submission and believe that I have an appropriate level of expertise to confirm that it is of an acceptable scientific standard.

# Reviewer report: Reviewer response for version 1

**DOI:** 10.21956/wellcomeopenres.22052.r74197

**Published:** 2024-03-27

**Authors:** Eileen Uribe-Querol

**Affiliations:** 1 Universidad Nacional Autónoma de México, Mexico City, Mexico

In the manuscript “Differential roles for the oxygen sensing enzymes PHD1 and PHD3 in the regulation of neutrophil metabolism and function, Watts and colleagues investigated the consequences of neutrophils oxygen sensing prolyl hydroxylase (PHD) enzymes, PHD1 and PHD3 deficiency in normoxia and hypoxia. While the authors addressed a clinically relevant issue and the data, character count, and number of figures make this manuscript a good candidate to be published, there are some limitations to their study and the findings need a more elaborated and clear mechanism of action. This topic is important because of the therapeutical use it can provide.

 Authors used a combination of in vivo and ex vivo models, utilizing neutrophil and myeloid specific PHD1 or PHD3 deficient mouse lines to investigate the roles of oxygen sensing prolyl hydroxylase enzymes in the regulation of neutrophilic inflammation and immunity and conclude that PHD1 deficiency drives alterations in neutrophil metabolism and recruitment, in an oxygen dependent fashion. On the other hand, PHD3 deficiency was found to enhance neutrophil antibacterial properties without excessive inflammatory responses.

 Mayor concerns:

 1) Methods section is not clear and incomplete. Each method should have a reference or a brief detailed description to allow replication. It is confusing were did neutrophils came from in some experiments. Please explain how blood and bone marrow neutrophils did were obtained. More information about the kits used is also needed.

 2) The papes has some interesting results that are not explained.

 3) Discussion section should be improved including a more elaborated description and analysis of the findings.

 Minor changes>

 1) All abbreviations need to be defined the first time mentioned on the paper.

 The paper must be proofread to correct typographic errors. Chemical formulas nomenclature and names should be reviewed. (O2), doble parenthesis, of some words that are not in context. In section, In vivo mouse models …”Pseudomonas aeruginosa 10”. What does the 10 stands for?

 2) Figure labels are two small and, in some cases, incomplete. All figures must be reviewed.

Is the work clearly and accurately presented and does it cite the current literature?

Partly

If applicable, is the statistical analysis and its interpretation appropriate?

Partly

Are all the source data underlying the results available to ensure full reproducibility?

Partly

Is the study design appropriate and is the work technically sound?

Partly

Are the conclusions drawn adequately supported by the results?

No

Are sufficient details of methods and analysis provided to allow replication by others?

No

Reviewer Expertise:

Neutrophil functions, developmental biology

I confirm that I have read this submission and believe that I have an appropriate level of expertise to confirm that it is of an acceptable scientific standard, however I have significant reservations, as outlined above.

# Response

**Published:** 2024-08-05

**Authors:** Emily Watts

**Affiliations:** Institute for Regeneration and Repair, The University of Edinburgh, Edinburgh, Scotland, UK

We would like to thank the reviewer for their time and their review of this manuscript. We have submitted these data to Wellcome Open Research as we wanted to share both the interesting positive findings, but also the important negative data, with the scientific community in a timely fashion. We agree that some of the findings are preliminary and that we have not fully dissected the mechanisms underlying the phenotypes which we have observed. Nevertheless, we felt that the phenotype observed in the PHD3 deficient mice was of sufficient interest to publish the data in its current form. Further studies will of course be required to confirm the mechanisms underpinning these findings. We are providing some limited new experimental data in this revised version but the majority of the comments raised have been addressed in the attached rebuttal and by revision of the text and conclusions drawn. We are grateful to the reviewer for their recognition of clinical relevance and of our work and hope that in this revised version they will agree that the conclusions drawn are supported by the data.

**In the manuscript “Differential roles for the oxygen sensing enzymes PHD1 and PHD3 in the regulation of neutrophil metabolism and function, Watts and colleagues investigated the consequences of neutrophils oxygen sensing prolyl hydroxylase (PHD) enzymes, PHD1 and PHD3 deficiency in normoxia and hypoxia. While the authors addressed a clinically relevant issue and the data, character count, and number of figures make this manuscript a good candidate to be published, there are some limitations to their study and the findings need a more elaborated and clear mechanism of action. This topic is important because of the therapeutical use it can provide.**

**Authors used a combination of in vivo and ex vivo models, utilizing neutrophil and myeloid specific PHD1 or PHD3 deficient mouse lines to investigate the roles of oxygen sensing prolyl hydroxylase enzymes in the regulation of neutrophilic inflammation and immunity and conclude that PHD1 deficiency drives alterations in neutrophil metabolism and recruitment, in an oxygen dependent fashion. On the other hand, PHD3 deficiency was found to enhance neutrophil antibacterial properties without excessive inflammatory responses.**

**Major concerns:**

**1) Methods section is not clear and incomplete. Each method should have a reference or a brief detailed description to allow replication. It is confusing were did neutrophils came from in some experiments. Please explain how blood and bone marrow neutrophils did were obtained. More information about the kits used is also needed.**

Response: Thank you for this feedback. We have added additional details to the methods section to describe in detail the neutrophil isolation protocols. For convenience these are also detailed here: Bone marrow: Both hind limbs were dissected out and stored in a clean bijou container on ice until processed. Each bone was flushed using 10ml of 1xHBSS with 0.2% BSA. The cell suspension was resuspended, passed through a 70mm filter into a fresh 50ml polystyrene falcon tube and then centrifuged at 450G for 10 minutes. 90% vol/vol percoll stock was made using 10X HBSS with NaHCO _3_ and this stock was used to make 81%, 62% and 55% percoll with 1x HBSS. All three percoll preparations were layered in a 15ml falcon tube and the cells were resuspended in 3ml of 1xHBSS with 0.2% BSA which was layered on top of the 52% percoll layer. The gradient was centrifuged at 2000G for 30 minutes (acceleration 1, deceleration 0). Bone marrow monocyte precursors were removed from the top layer with a pastette. Neutrophils were removed from the middle layer and washed in 30ml of 1xHBSS with 0.2% BSA. Hypotonic saline red blood cell lysis was carried out. Following the percoll purification above, neutrophil purity was found to be approximately 90%. In order to generate highly pure bone marrow neutrophils samples, cells from the neutrophil layer were resuspended in 1xHBSS with 0.2% BSA and purified by fluorescence-activated cell sorting (FACS) based on the forward/side scatter profile and auto-fluorescence as previously described ^1^. Bronchoalveolar lavage: Where highly pure BAL neutrophils were required, we used a previously optimized discontinuous percoll gradient coupled with a red cell lysis step in order to gain purities of >98%. BAL cells were first pelleted by centrifugation at 350G for 10 minutes. BAL samples from 1-3 mice (depending on the final cell number required) were pooled onto a single percoll gradient. 90% vol/vol percoll was made using 10X PBS and further percoll solutions were made using this stock 90% percoll mixed with 1X PBS. 3ml of 78% percoll was pipetted into the bottom of a 15ml falcon tube, followed by 3ml of 69% percoll. The cell pellet(s) were resuspended in 3ml of 52% percoll which was then layered onto the 69% layer. The gradient(s) were centrifuged at 1200G for 30mins with an acceleration of 1 and deceleration of 0. Following centrifugation, the macrophages accumulated on the top of the 52% layer and were removed using a sterile pastette. The neutrophils (within the 69% layer) were then removed using a fresh sterile pastette and placed into a 50ml polystyrene falcon tube. This was topped up to 50ml with sterile PBS to wash off the percoll and the purified neutrophils pelleted at 350G for 8 mins. Residual red cells were lysed by hypotonic saline lysis. A cytospin was made prior to the final centrifugation step to assess purity by morphology. During optimisation of this protocol, purity was also confirmed by flow cytometry and the neutrophils were found to be >98% pure.

**2) The paper has some interesting results that are not explained.**

Response: We agree that this paper does not fully dissect the mechanisms underlying the observed phenotypes and have provided a more detailed explanation in response to reviewers comments (please see separate responses). We have published these results (including relevant negative data) in Wellcome Open Reports to ensure timely availability of these data to the wider community. We have expanded the discussion and limitations sections to acknowledge clearly that these data are preliminary findings rather than definitive mechanistic data and hope that by sharing the data, further work can be done to interrogate the interesting findings in more detail.

**3) Discussion section should be improved including a more elaborated description and analysis of the findings.**

Response: As above, taking in account comments from the reviewers we have now expanded the discussion and limitations sections to better describe the findings and to address specific queries.

**Minor changes**

**1) All abbreviations need to be defined the first time mentioned on the paper.**

**The paper must be proofread to correct typographic errors. Chemical formulas nomenclature and names should be reviewed. (O2), doble parenthesis, of some words that are not in context. In section, In vivo mouse models …”Pseudomonas aeruginosa 10”. What does the 10 stands for?**

Response: Thank you. We have corrected the errors detailed. The LPS is sourced from *Pseudomonas aeruginosa* (10) ( https://www.sigmaaldrich.com/GB/en/product/sigma/l9143). We apologise that the omission of the brackets may have made this unclear and have updated the manuscript for clarity.

**2) Figure labels are two small and, in some cases, incomplete. All figures must be reviewed.** 

Response: Thank you. We have increased the size of the figure labels. All figures were submitted at equal resolution and this technical issue has been highlighted to the editorial team and will be resolved in the final version.

 1. Dorward, D. A. *et al.* Technical advance: autofluorescence-based sorting: rapid and nonperturbing isolation of ultrapure neutrophils to determine cytokine production. *Journal of Leukocyte Biology* **94,** 193–202 (2013).

# Reviewer report: Reviewer response for version 1

**DOI:** 10.21956/wellcomeopenres.22052.r74200

**Published:** 2024-03-12

**Authors:** Frederik Denorme

**Affiliations:** 1 Emergency Medicine, Washington University in St Louis School of Medicine, St. Louis, Missouri, USA

In this article, Watss and colleagues investigate the role of neutrophil PHD1 and 3 in murine models of inflammation. The study is interesting, however, some findings are rather preliminary due to low N. Additionally, the authors tend to over-interpret their data, drawing somewhat strong conclusions from a rather minor phenotype. 

 - For the in vitro assays, neutrophils were isolated. However, it is nowhere specified how they were isolated and how purity was validated. 

 - NET formation assays are also not specified. What agonist was used to induce NET formation and for how long?

 - Since these are new mice that have been generated, I am surprised no more baseline characterisation was performed. I.e. on neutrophils isolated in the absence of an inflammatory trigger. 

 - How was sample size determined for the experiments? It seems to vary a lot based on Figure and doesn't seem to make much sense. Was there a power analysis? For example Fig; 1C seems to be trending or close to significance, yet there are only 3 samples per group and no P-value is provide. While some other assays show significance with smaller differences, however, bigger sample size. This becomes somewhat concerning if the authors try to draw mechanistic conclusions from these results. 

 - Another way to interpret Figure 1H-I, is that there are less neutrophils being recruited under hypoxia, and that this reduction is abrogated in the KO mice. 

 - What about neutrophil resolution in normoxia in both groups?

 - What do the authors hypothesise is the reason for similar NE but different neutrophil numbers in mice. If there are more neutrophils but they don't get activated as well, wouldn't the netto impact be no difference. 

 - Figure 3: reduced neutrophil numbers are rather minor, as half of the WT mice are in the same range of KO animals. Similar for Figure 4, the differences are rather minor, the conclusions need to be toned down a little bit. The biggest difference seems to be with CFU at day 2, however this difference disappears over time. 

 - Figure 5 fits with the rest of the manuscript as there are some small differences. The title reduced bacterial load seems a little over-reaching since the difference is only in BAL and only really 4/10 WT mice have more bacteria than the KO mice. 

 - Figure 6A could be an explanation for a lot of the previous data, however only an N of 3 is done. This would need to be increased. It is also unclear if a paired t-test is the best statistical test for this type of assay.

Is the work clearly and accurately presented and does it cite the current literature?

Yes

If applicable, is the statistical analysis and its interpretation appropriate?

Yes

Are all the source data underlying the results available to ensure full reproducibility?

Yes

Is the study design appropriate and is the work technically sound?

Yes

Are the conclusions drawn adequately supported by the results?

Partly

Are sufficient details of methods and analysis provided to allow replication by others?

Yes

Reviewer Expertise:

Neutrophil biology in the context of (sterile) inflammation.

I confirm that I have read this submission and believe that I have an appropriate level of expertise to confirm that it is of an acceptable scientific standard, however I have significant reservations, as outlined above.

# Response

**Published:** 2024-08-05

**Authors:** Emily Watts

**Affiliations:** Institute for Regeneration and Repair, The University of Edinburgh, Edinburgh, Scotland, UK

We would like to thank the reviewer for their time and their detailed review of this manuscript. We have submitted these data to Wellcome Open Research as we wanted to share both the interesting positive findings, but also the important negative data, with the scientific community in a timely fashion. We agree that some of the findings are preliminary and that we have not fully dissected the mechanisms underlying the phenotypes which we have observed. Nevertheless, we felt that the phenotype observed in the PHD3 deficient mice was of sufficient interest to publish the data in its current form. Further studies will of course be required to confirm the mechanisms underpinning these findings. We are providing some limited new experimental data in this revised version but the majority of the comments raised have been addressed in the attached rebuttal and by revision of the text and conclusions drawn. We are grateful to the reviewer for their recognition of the interesting nature of our work and hope that in this revised version they will agree that the conclusions drawn are supported by the data.

**In this article, Watts and colleagues investigate the role of neutrophil PHD1 and 3 in murine models of inflammation. The study is interesting, however, some findings are rather preliminary due to low N. Additionally, the authors tend to over-interpret their data, drawing somewhat strong conclusions from a rather minor phenotype.** **- For the in vitro assays, neutrophils were isolated. However, it is nowhere specified how they were isolated and how purity was validated.**

Response: We apologise for this oversight. We have now updated the methods section to include the detailed protocols for neutrophil isolation and have included them below for convenience: Bone marrow: Both hind limbs were dissected out and stored in a clean bijou container on ice until processed. Each bone was flushed using 10ml of 1xHBSS with 0.2% BSA. The cell suspension was resuspended, passed through a 70mm filter into a fresh 50ml polystyrene falcon tube and then centrifuged at 450G for 10 minutes. 90% vol/vol percoll stock was made using 10X HBSS with NaHCO _3_ and this stock was used to make 81%, 62% and 55% percoll with 1x HBSS. All three percoll preparations were layered in a 15ml falcon tube and the cells were resuspended in 3ml of 1xHBSS with 0.2% BSA which was layered on top of the 52% percoll layer. The gradient was centrifuged at 2000G for 30 minutes (acceleration 1, deceleration 0). Bone marrow monocyte precursors were removed from the top layer with a pastette. Neutrophils were removed from the middle layer and washed in 30ml of 1xHBSS with 0.2%BSA. Hypotonic saline red blood cell lysis was carried out. Following the percoll purification above, neutrophil purity was found to be approximately 90%. In order to generate highly pure bone marrow neutrophils samples, cells from the neutrophil layer were resuspended in 1xHBSS with 0.2% BSA and purified by fluorescence-activated cell sorting (FACS) based on the forward/side scatter profile and auto-fluorescence as previously described ^1^ Bronchoalveolar lavage: Where highly pure BAL neutrophils were required, we used a previously optimized discontinuous percoll gradient coupled with a red cell lysis step in order to gain purities of >98%.  BAL cells were first pelleted by centrifugation at 350G for 10 minutes. BAL samples from 1-3 mice (depending on the final cell number required) were pooled onto a single percoll gradient. 90% vol/vol percoll was made using 10X PBS and further percoll solutions were made using this stock 90% percoll mixed with 1X PBS. 3ml of 78% percoll was pipetted into the bottom of a 15ml falcon tube, followed by 3ml of 69% percoll. The cell pellet(s) were resuspended in 3ml of 52% percoll which was then layered onto the 69% layer. The gradient(s) were centrifuged at 1200G for 30mins with an acceleration of 1 and deceleration of 0. Following centrifugation, the macrophages accumulated on the top of the 52% layer and were removed using a sterile pastette. The neutrophils (within the 69% layer) were then removed using a fresh sterile pastette and placed into a 50ml polystyrene falcon tube. This was topped up to 50ml with sterile PBS to wash off the percoll and the purified neutrophils pelleted at 350G for 8 mins. Residual red cells were lysed by hypotonic saline lysis. A cytospin was made prior to the final centrifugation step to assess purity by morphology. During optimisation of this protocol, purity was also confirmed by flow cytometry and the neutrophils were found to be >98% pure.  

**NET formation assays are also not specified. What agonist was used to induce NET formation and for how long?**

Response: The NET assay was carried out on *ex-vivo* BAL neutrophils following LPS induced lung injury, therefore highly activated cells. We did not further stimulate them (for example with PMA) but instead measured baseline NETosis using SyTox Red. We apologise for the lack of clarity in the description of this assay and have reworded it to improve this.

**Since these are new mice that have been generated, I am surprised no more baseline characterisation was performed. I.e. on neutrophils isolated in the absence of an inflammatory trigger.**

Response: The PHD3 ^fl/fl^LysMCre ^+/- ^mouse line has been previously published ^2^. MRP8 driven Cre is established as a method of inducing neutrophil specific knock down ^3-6^. In this revised version we provide additional data to show normal circulating leucocyte populations in naïve mice from both PHD1 ^fl/fl^MRP8Cre ^+/-^ PHD3 ^fl/fl^MRP8Cre ^+/- ^mice. We also demonstrate effective expression of the Cre recombinase using it’s GFP tag in circulating neutrophils.

**How was sample size determined for the experiments? It seems to vary a lot based on Figure and doesn't seem to make much sense. Was there a power analysis? For example Fig; 1C seems to be trending or close to significance, yet there are only 3 samples per group and no P-value is provide. While some other assays show significance with smaller differences, however, bigger sample size. This becomes somewhat concerning if the authors try to draw mechanistic conclusions from these results.**

Response: Sample size was determined by a number of factors including our previous experience using these models and the degree of biological variability which is seen. For example, in our experience, there is significant variability in response to the streptococcal pneumonia model and so we planned to use a higher sample size in these experiments. In some assays, such as the metabolite mass spec data sets and in the PHD3 *in vitro *bacterial killing, each sample represents cells pooled from up to 3 mice and so sample size was kept to a minimum to avoid excessive use of animals. We agree that there is a trend towards reduced PHD1 expression in the hypoxic BAL neutrophils and it may be informative to repeat this to see if it does reach significance, the p value here is 0.1010 with n=3. In light of the predominantly negative data we found in the PHD1 mice, we have elected not to repeat these experiments at present as it is unlikely to alter our immediate future work but accept the reviewer’s comments regarding this figure.

**Another way to interpret Figure 1H-I, is that there are less neutrophils being recruited under hypoxia, and that this reduction is abrogated in the KO mice.**

Response: Thank you for this interesting comment. We do consistently see a small reduction in neutrophil recruitment to the airways in hypoxia (see Watts *et al.* ^7^ Fig 6D). We suggest that our data here shows that the increased rate of resolution seen in the hypoxic PHD1 mice is a result of both higher numbers at 24 hours and lower numbers at 48 hours when compared to wild type animals. We have updated the text in the manuscript to better describe this phenotype.

**What about neutrophil resolution in normoxia in both groups?**  

Response: Resolution is not altered in the PHD1 deficient mice in normoxia, we have now included this data in the manuscript for completeness (Fig 1M).

**What do the authors hypothesise is the reason for similar NE but different neutrophil numbers in mice. If there are more neutrophils but they don't get activated as well, wouldn't the net impact be no difference.**

Response: Thank you for this interesting point. We would have to conclude that the PHD1 deficient neutrophils are producing less elastase than their wild-type counter parts. Indeed, if one calculates the “elastase per neutrophil” and then the fold change from WT to KO, this is ~0.85. Nevertheless, the overall impact of these changes is not significant as we don’t see any difference in lung injury (i.e. albumin leak) or in physiological parameters such a temperature. These data are in keeping with our overarching conclusion that, although PHD1 deficiency does have some effects on neutrophil function, it is not of sufficient magnitude to result in a significant phenotype overall. We have expanded on the existing text to better describe these findings.

**Figure 3: reduced neutrophil numbers are rather minor, as half of the WT mice are in the same range of KO animals. Similar for Figure 4, the differences are rather minor, the conclusions need to be toned down a little bit. The biggest difference seems to be with CFU at day 2, however this difference disappears over time.**

Response: With regards to figure 3D, there is overlap between the PHD3 ^fl/fl^LysMCre ^-/-^ and PHD3 ^fl/fl^LysMCre ^+/-^ groups, in keeping with biological variability between animals in response to the LPS induced lung injury. However, the mean neutrophil number for PHD3 ^fl/fl^LysMCre ^-/-^ is 7.28 million versus 4.60 million in the PHD3 ^fl/fl^LysMCre ^-/+^, a fold change of 0.63 which we consider to be both a biologically and statistically significant reduction in neutrophil cell number. We accept that the amplitude of the changes in Figure 4 are small in some cases. The significant difference in CFU counts at day 2 is important as it likely leads to the overall smaller abscess size over the course of the 7 days course. We have reworded the conclusions as suggested in order to avoid overstating the size of the effect.

**Figure 5 fits with the rest of the manuscript as there are some small differences. The title reduced bacterial load seems a little over-reaching since the difference is only in BAL and only really 4/10 WT mice have more bacteria than the KO mice.**

Response: The scale on this graph is logarithmic which does make it difficult to appreciate the difference in the groups. As before, there is significant variability between mice in both groups but the fold change is from PHD3 ^fl/fl^LysMCre ^-/-^ to PHD3 ^fl/fl^LysMCre ^+/-^ groups is 0.02 and reaches statistical significance. These data are in keeping with our other *in vivo* and *in vitro* findings so we do feel that they are valid and important results.

**Figure 6A could be an explanation for a lot of the previous data, however only an N of 3 is done. This would need to be increased. It is also unclear if a paired t-test is the best statistical test for this type of assay.** 

Response: We accept that this is a small N number. However, each N is generated by pooling the BAL cells from 3-4 mice (per genotype) in order to generate enough cells to run in duplicate and at multiple dilutions for CFU counts. Given that we are able to see a small but significant difference with the data we have generated we didn’t feel it was justifiable within the 3Rs framework to repeat this assay simply to increase numbers. Within our group we have carried out this assay in multiple mouse lines and in human peripheral blood neutrophils and found the data to be normally distributed, justifying the use of a t-test here. There is a degree of variability between runs e.g. due to differences in aliquots of bacteria used and so we have used a paired t-test. We could also have presented this data as fold change from WT in each run but felt that the raw data was a more complete and transparent way to present the findings.

 1. Dorward, D. A. *et al.* Technical advance: autofluorescence-based sorting: rapid and nonperturbing isolation of ultrapure neutrophils to determine cytokine production. *Journal of Leukocyte Biology* **94,** 193–202 (2013).

 2. Swain, L. *et al.* Prolyl-4-hydroxylase domain 3 (PHD3) is a critical terminator for cell survival of macrophages under stress conditions. *Journal of Leukocyte Biology* **96,** 365–375 (2014).

 3. Singhal, R. *et al.* Disruption of hypoxia-inducible factor-2α in neutrophils decreases colitis-associated colon cancer. *Am J Physiol Gastrointest Liver Physiol* **326,** G53–G66 (2024).

 4. Patel, R. B. *et al.* Targeting Neutrophil α9 Improves Functional Outcomes After Stroke in Mice With Obesity-Induced Hyperglycemia. *Stroke* **54,** 2409–2419 (2023).

 5. Idol, R. A. *et al.* Neutrophil and Macrophage NADPH Oxidase 2 Differentially Control Responses to Inflammation and to Aspergillus fumigatus in Mice. *J. Immunol.* **209,** 1960–1972 (2022).

 6. Elliott, E. R. *et al.* Deletion of Syk in Neutrophils Prevents Immune Complex Arthritis. *The Journal of Immunology* **187,** 4319–4330 (2011). 7.     Watts, E. R. *et al.* Hypoxia drives murine neutrophil protein scavenging to maintain central carbon metabolism. *J. Clin. Invest.* **131,** (2021).

# Reviewer report: Reviewer response for version 1

**DOI:** 10.21956/wellcomeopenres.22052.r74196

**Published:** 2024-03-04

**Authors:** Nathachit Limjunyawong

**Affiliations:** 1 Mahidol University, Bangkok, Bangkok, Thailand

In this study by Watts et al., the researchers have expanded upon their previous work wherein they investigated the expressions and roles of three isoforms of prolyl hydroxylase domain (PHD) enzymes - namely, PHD1, PHD2, and PHD3 - within neutrophils *. *Previously, their focus centered on PHD2, revealing its constitutive expression in neutrophils with a potential for upregulation during sterile inflammation. The deletion of PHD2 specifically within the myeloid population was found to potentially exacerbate inflammatory response to bacterial infection by augmenting glycolytic flux, thereby enhancing neutrophil functional capacity. However, myeloid-specific PHD2 deficiency did not amplify antibacterial properties *. *In this current study, the authors further examined the significance of the other two isoforms of PHD enzymes in neutrophils, elucidating their distinct functions. Although PHD1 deficiency in neutrophils was found to alter oxygen-dependent metabolic profiles and the recruitment of neutrophils, it did not affect neutrophil functional capacity either *ex vivo* or *in vivo*.  On the other hand, the depletion of PHD3 in myeloid cells resulted in enhanced bactericidal capacity, potentially through increased oxygen consumption and mitochondrial reactive oxygen species generation.

 While the study reveals intriguing findings, it is imperative to address specific comments and concerns to bolster the robustness of the results and improve clarity. The primary concern lies in the lack of a clear rationale and coherence between the studies, making it challenging to comprehend the authors' transitions from one experiment to another. For instance:

- The rationale behind the switch in Cre lines from MRP8-Cre for PHD1 deletion to LysM-Cre for PHD3 deletion remains unclear, leading to difficulties in comparing the outcomes of gene deficiency, particularly considering the discrepancy in patterns of expression of these two promoter-driven deletions. While the MRP8-Cre is by far the most neutrophil-specific Cre line, the LysM-Cre targets several myeloid cell types, posing challenges in isolating the effects solely on neutrophils. Although the authors acknowledged in the Limitations that both neutrophil-specific and myeloid-specific mouse lines in the PHD3 were used, only myeloid-specific deletion data were presented in this study. Moreover, the mice used in this study were newly generated and involved the crossing of different lines together (e.g., crossing PHD1fl/fl animals with MRP8-Cre-ires/GFP mice). The evidence showing the successful deletion of the interested gene only in anticipated specific cell types such as neutrophils, but not other related immune cells is required. In particular, it is crucial to ascertain whether the expression of PHD3 gene was absent only in neutrophils or if it was also affected in other myeloid cells such as alveolar macrophages in PHD3 ^fl/fl^LysMCre ^+/-^ mice. And how would the authors explain all the results if the expression of the PHD3 gene was altered in other cell types as well? Figure 3A shows the upregulation of PHD3 expression in BAL neutrophils after LPS-induced lung injury in hypoxia conditions, but were there upregulation in other non-neutrophil myeloid cells as well? Additionally, assessing HIF level/activity or its downstream of PHD/HIF axis in the immune cells isolated from these transgenic animals could provide insight into the association of the results with the oxygen sensing pathway, thereby strengthening the findings.
- It is unclear why the authors mainly utilized the LPS+normoxia/hypoxia model (but no infection model at all) in studying the role of PHD1, whereas the LPS+normoxia model (without hypoxia condition) was only briefly used prior to shifting focus to primarily investigate infection models for PHD3 study.
- Several inconsistencies in experimental designs across different studies are noticed without providing adequate reasons. For example, although metabolic profiles were obtained in both studies, the Seahorse experiments were exclusively conducted in PHD3 studies, while energy status measurement (ATP/ADP level) was only conducted in the PHD1 study. Moreover, certain assays such as MPO or NETosis were not performed in the PHD1 *ex vivo* study, whereas chemotaxis was not observed in the PHD3 *ex vivo* study. Additionally, while neutrophil degranulation (e.g., MPO and elastase concentration) was evaluated mainly in *in vivo* studies, it was not assessed in *ex vivo* studies at all. The BAL albumin was used to assess vascular leakage in the lung for sterile inflammation in Fig. 3, but the BAL IgM was obtained to evaluate vascular leakage in the lung for bacterial infection in Fig. 5.

Throughout the manuscripts, the authors neglected to include control groups in most experiments to assess whether the deficiency of the gene impacted baseline parameters. For example, it remains unclear whether PHD1 ^fl/fl^MRP8Cre ^+/-^ mice exhibited neutrophil numbers at the baseline comparable to PHD1 ^fl/fl^MRP8Cre ^-/-^ control animals in Fig. 1, or whether the absence of PHD1 in neutrophils altered the oxygen consumption rate or any metabolic state of neutrophils at the baseline (without LPS treatment) in Fig. 2?

 Figure 1 is somewhat ambiguous. The sole difference observed between PHD1 ^fl/fl^MRP8Cre ^+/- ^vs PHD1 ^fl/fl^MRP8Cre ^-/-^ was a slight increase in neutrophils recruitment in PHD1 ^fl/fl^MRP8Cre ^+/- ^mice after LPS-induced lung injury in hypoxia. However, this conclusion was drawn without knowledge of the basal lung neutrophil levels of these two strains. The title of Fig. 1L, specified as the “resolution” of neutrophils numbers, was confusing as it was uncertain if the value indeed reflected the resolution (PHD1 ^fl/fl^MRP8Cre ^+/- ^mice appear to have better resolution, yet the value displayed was lower). The issue could be raised from the finding that the levels of BAL neutrophils in PHD1 ^fl/fl^MRP8Cre ^-/-^ mice remained unchanged between 24 hours and 48 hours (Fig. 1I and 1K), with no identified basal level of lung neutrophil number. This could be attributed to the possibility that the neutrophils were not recruited to the lungs after all the challenges in these PHD1 ^fl/fl^MRP8Cre ^-/-^ mice, thus obviating the need for resolution.

 Additionally, concerning Fig. 1, it is unclear why the authors opted to segregate the graphs displaying the results of normoxia (Fig. 1E-1G, Fig. 1M-1N) vs hypoxia (Fig. 1H-1J, Fig. 1O-1P) at 24 hours while merging both normoxia and hypoxia conditions for the 48-hours studies (Fig. 1K and Fig. 1Q). The concerns were raised considering the different statistical analysis used for 24 hours (Mann-Whitney test) vs 48 hours (ordinary one-way ANOVA).

  Although the authors illustrated that the expression of PHD3, but not PHD1 and PHD2, was significantly upregulated in neutrophils isolated from animals maintained in hypoxia (Figure 1C, 3A), none of the experiments in this manuscript explore the role of PHD3 in hypoxic conditions. Despite investigating the effects of PHD1 deficiency in neutrophils under both normoxic and hypoxic conditions, it is unexpected that all subsequent experiments regarding PHD3 functions were conducted using PHD3 ^fl/fl^LysMCre ^+/-^ mice solely in normoxia.

 Regarding the pneumococcal infection model depicted in Fig.5, clarification is needed regarding why the authors examined colony-forming units (CFU) only 14 hours after infection, a time point that may be too early to observe differences in terms of neutrophil function/recruitment (again, no baseline level of neutrophil number is provided, thus it is difficult to assess recruitment). At this early stage, it is worth considering whether the difference in CFU could potentially be attributed to the depletion of PHD3 in the other resident myeloid cells such as alveolar macrophages?

 A number of aspects of the Methods section and experimental design require careful attention. Detailed protocols for several parts are notably absent.

- Most *in vitro* studies in the manuscript relied on either isolated bone marrow neutrophils or isolated BAL neutrophils. However, the Methods section lacks comprehensive information on how to isolate and purify these cells. While it was mentioned that “Bone marrow neutrophils were isolated by percoll gradient followed by fluorescence activated cell sorting to ensure sufficient purity.” in the legend of Fig. 1, specific details such as how to perform percoll gradient, whether BAL samples underwent percoll gradient enrichment, or the FACS sorting process, including the markers used to label neutrophils, were not provided in the Methods section. The authors should also mention if the purity of the neutrophils post-purification was confirmed.
- In the “LPS lung injury” section: details regarding the conditions/treatments used as control, such as nebulizing the animals with PBS, or the method of inducing normoxia compared to hypoxia, are lacking. Also, in the statement: “Mice were treated with lipopolysaccharide (LPS) from *Pseudomonas aeruginosa* **10**”, not sure what “10” means?
- The Methods in “Phagocytosis” section lack details of how flow cytometry was employed to assess phagocytosis and obtain **%**phagocytosis (in Fig. 2K, 6B, 6C). The term "ice control" shown in the X-axis of Fig. 2K requires explanation. Including representative flow cytometry analysis figures of phagocytosis could enhance clarity. Moreover, while the method section specifies the use of “fluorescently labelled, heat-killed *E. Coli* (Invitrogen)” for the study which I believe shown in Fig. 2K, it did not mention the Staphylococcus and Streptococcus strains used in the phagocytosis assay in Fig. 6B and 6C. These should be mentioned in the Methods section as well.
- For NETosis assay, more details are needed on how Sytox Red could be used to assess NET formation and NETosis by flow cytometry. It is unclear what percentage of NETosis means. What was the control of this NETosis study? Were isolated neutrophils challenged with any bacteria/insults to stimulate the release of NETs before fixing the cells with PFA?
- Regarding the respiratory burst assay, the authors used 10 uM fMLP to induce a respiratory burst. However, considering the authors’ previous work [Fig. 2, Sadiku P, et. al. 2017 (Ref 1)], 10uM of fMLP did not activate respiratory burst signal in BAL neutrophils isolated from WT animals. An explanation for this discrepancy between the two studies is warranted. Also, the abbreviation "fMLF" in the Methods section should be changed to "fMLP" for consistency with the abbreviation used in Fig. 2.
- The specification of negative control and positive control for the chemotaxis assay shown in Fig. 2J, along with clarification on how to obtain %positive control, is necessary.
- The authors should provide details on how they analyzed qPCR results to determine relative gene expression. Were they reported as 2^-(ddCT) or just normalized to *Actb* expression? If analyzed using the 2^-(ddCT) method, it is unclear what sample was used as a reference for normalization, as none of the values in Fig. 1A were close to 1. Conversely, if the results were relative to *Actb* expression, it is difficult to believe that PHD3 expression was upregulated to a level 20-fold higher than *Actb* expression. Confirmation of these findings and the methodology used for analysis is necessary.
- Further elaboration on Seahorse experiments (Fig. 6E-6J) should be included in the Methods section. This includes information on when compounds (glucose, oligo, 2DG) were added to the cells, and at what concentrations, as well as the time point after bacterial infection shown in Fig. 6F and 6I (before or after adding other compounds). Also, did these compound treatments facilitate the explanation of any findings of the story?
- Please verify why the authors used 2-way ANOVA for metabolic profile analysis depicted in Fig. 2A-2F.

Considering the findings on metabolic activity, it would be beneficial to provide an explanation or discussion on why metabolites in the OxPPP flux were reduced in PHD1 ^fl/fl^MRP8Cre ^+/- ^mice specifically under normoxic conditions (Fig. 2B), while metabolites in TCA cycles were increased in these mice only under hypoxia condition (Fig. 2F).

 Furthermore, in Fig. 6G, there appears to be a trend toward a higher oxygen consumption rate (OCR) in PHD3 ^fl/fl^MRP8Cre ^+/- ^mice even at baseline before bacterial infection. It would be valuable for the authors to address this observation. Additionally, although there was an increase in OCR in these PHD3-depleted neutrophils, no changes were observed in any metabolite intermediates, including those in the OxPPP pathway (Fig. 6M). It would be helpful if the authors could provide an explanation for this discrepancy.

 Minor concerns:

- Please ensure to define all the abbreviations the first time mentioned in the manuscript e.g., IVC, BAL, IT, CFU, MPO, 2DG, all metabolites (e.g., F6P, F16BP, GAO, PEP, etc.)
- Although the interested gene was intact in these animals, I suggest avoiding to use of the term “wildtype” for all the floxed control animals.
- Methods in “Assessing bacterial load/CFU counts” section: Suggest changing “bijou” to “Bijou container”
- Methods in “Assessment of MPO activity in Staphylococcus aureus skin abscess model” section: Please make sure to italicize *Staphylococcus aureus*
- Methods in “CFSE labelling of *Streptococus pneumoniae*” section: should be “ *Streptococcus*”
- Please make sure to write the correct format for all mRNA/protein nomenclature with consistency throughout the manuscript. For example, RNA level (mouse) should be written italicized as “ *Egln1*”
- Figure 1A: please make sure the title is labeled as “PHD2” (looks like PI-D2 in the pdf file), and also label the Y-axis with the gene name in Fig. 1C to be consistent with Fig. 1A, 1B
- Please change the Y-axis label in Fig.2A, 2D to be similar to Fig. 2B-2C or Fig. 2E-2F
- Figure 2I-2K needs labels for what black vs gray dots represent. Also, it should be DCF (“ **I”**), chemotaxis (“ **J”**), and phagocytosis (“ **K”**) in the figure legend
- The authors stated that “ex-vivo BAL cells from the neutrophil specific PHD3fl/flMRP8Cre+/- mouse lines with *Staphylococcus aureus*. We used glucose **-depleted** media to represent the hypoglycaemic airway”. This is confusing since the authors used *Staphylococcus aureus* for the skin infection model, not the airway infection model.
- Figure 4A requires better-quality images
- Figure 4E needs labels for what black vs white dots represent. What was the time-point after infection of this finding? And what did the p-value mean here?
- Fig. 4F-4H: why did the authors normalize the CFU per abscess instead of per gram as shown in Fig. 4E? And it is surprising to see that CFU was increased over time from day 2 to day 7. Does this normally occur in this staphylococcal skin infection model?
- Fig. 5E: what is “SN” in the title of the figure?
- Fig. 6A: more N should be added to confirm the findings.
- Fig. 6E, 6H: please ensure species names were written in the correct format ( *S. aureus*)

Is the work clearly and accurately presented and does it cite the current literature?

Yes

If applicable, is the statistical analysis and its interpretation appropriate?

Partly

Are all the source data underlying the results available to ensure full reproducibility?

Yes

Is the study design appropriate and is the work technically sound?

Partly

Are the conclusions drawn adequately supported by the results?

Partly

Are sufficient details of methods and analysis provided to allow replication by others?

No

Reviewer Expertise:

Innate immunity, Allergy, Immunology, Infectious diseases, Pulmonary diseases, MRGPR, Mast cells

I confirm that I have read this submission and believe that I have an appropriate level of expertise to confirm that it is of an acceptable scientific standard, however I have significant reservations, as outlined above.

## References

[1] : Prolyl hydroxylase 2 inactivation enhances glycogen storage and promotes excessive neutrophilic responses. *J Clin Invest* .2017;127(9) : 10.1172/JCI90848 3407-3420 10.1172/JCI90848 28805660 PMC5669581

# Response

**Published:** 2024-08-05

**Authors:** Emily Watts

**Affiliations:** Institute for Regeneration and Repair, The University of Edinburgh, Edinburgh, Scotland, UK

We would like to thank the reviewer for their time and their detailed review of this manuscript. We have submitted these data to Wellcome Open Research as we wanted to share both the interesting positive findings, but also the important negative data, with the scientific community in a timely fashion. We agree that some of the findings are preliminary and that we have not fully dissected the mechanisms underlying the phenotypes which we have observed. Nevertheless, we felt that the phenotype observed in the PHD3 deficient mice was of sufficient interest to publish the data in its current form. Further studies will of course be required to confirm the mechanisms underpinning these findings. We are providing some limited new experimental data in this revised version but the majority of the comments raised have been addressed in the attached rebuttal and by revision of the text and conclusions drawn. We are grateful to the reviewer for their recognition of the interesting nature of our findings and hope that in this revised version they will agree that the conclusions drawn are supported by the data.

**In this study by Watts et al., the researchers have expanded upon their previous work wherein they investigated the expressions and roles of three isoforms of prolyl hydroxylase domain (PHD) enzymes - namely, PHD1, PHD2, and PHD3 - within neutrophils *. *Previously, their focus centered on PHD2, revealing its constitutive expression in neutrophils with a potential for upregulation during sterile inflammation. The deletion of PHD2 specifically within the myeloid population was found to potentially exacerbate inflammatory response to bacterial infection by augmenting glycolytic flux, thereby enhancing neutrophil functional capacity. However, myeloid-specific PHD2 deficiency did not amplify antibacterial properties *. *In this current study, the authors further examined the significance of the other two isoforms of PHD enzymes in neutrophils, elucidating their distinct functions. Although PHD1 deficiency in neutrophils was found to alter oxygen-dependent metabolic profiles and the recruitment of neutrophils, it did not affect neutrophil functional capacity either *ex vivo* or *in vivo*.  On the other hand, the depletion of PHD3 in myeloid cells resulted in enhanced bactericidal capacity, potentially through increased oxygen consumption and mitochondrial reactive oxygen species generation.**

**While the study reveals intriguing findings, it is imperative to address specific comments and concerns to bolster the robustness of the results and improve clarity. The primary concern lies in the lack of a clear rationale and coherence between the studies, making it challenging to comprehend the authors' transitions from one experiment to another. For instance:** **-The rationale behind the switch in Cre lines from MRP8-Cre for PHD1 deletion to LysM-Cre for PHD3 deletion remains unclear, leading to difficulties in comparing the outcomes of gene deficiency, particularly considering the discrepancy in patterns of expression of these two promoter-driven deletions. While the MRP8-Cre is by far the most neutrophil-specific Cre line, the LysM-Cre targets several myeloid cell types, posing challenges in isolating the effects solely on neutrophils. Although the authors acknowledged in the Limitations that both neutrophil-specific and myeloid-specific mouse lines in the PHD3 were used, only myeloid-specific deletion data were presented in this study.**

Response: We have presented both myeloid and neutrophil specific PHD3 data in this study, a number of the figures in Figure 6 are carried out in the MRP8, neutrophil specific line, as per the axis labels. We accept that there are challenges in comparing the data between these different Cre lines due to their different specificities but felt that the PHD1 data was important negative data which we wished to make available to other researchers working in the field of hypoxia. This work was carried out separately from the PHD3 work and our aim in this paper was not necessarily to directly compare PHD1 with PHD3 deficiency, rather to present the data from both genotypes for interest and completeness. We have reworded the conclusions and limitations sections in order to improve clarity and to better explain the use of the different mouse lines.

**Moreover, the mice used in this study were newly generated and involved the crossing of different lines together (e.g., crossing PHD1fl/fl animals with MRP8-Cre-ires/GFP mice). The evidence showing the successful deletion of the interested gene only in anticipated specific cell types such as neutrophils, but not other related immune cells is required. In particular, it is crucial to ascertain whether the expression of PHD3 gene was absent only in neutrophils or if it was also affected in other myeloid cells such as alveolar macrophages in PHD3 ^fl/fl^LysMCre ^+/-^ mice. ** 

Response: MRP8 driven Cre is established as a method of inducing neutrophil specific knock down ^1-4^. We now provide additional data to demonstrate effective expression of the Cre recombinase using it’s GFP tag in circulating neutrophils (Figure 1B and 6B in the revised manuscript).

**And how would the authors explain all the results if the expression of the PHD3 gene was altered in other cell types as well? Figure 3A shows the upregulation of PHD3 expression in BAL neutrophils after LPS-induced lung injury in hypoxia conditions, but were there upregulation in other non-neutrophil myeloid cells as well?**

Response: PHD3 is likely to be regulated in other cells types in hypoxia. The PHD3 data in this manuscript only relates to normoxic conditions. We have previously published on PHD3 deficiency in hypoxic neutrophils ^5^ and further characterisation of the impact of PHD3 loss on hypoxic conditions is of course of great interest but beyond the scope of this article. We have not looked at regulation of PHD3 by hypoxia in other immune cells but others have shown that PHD3 is upregulated in response to hypoxia in a wide range of cell lines and primary cells, including in the macrophage/monocytes compartment ^6,7^

**Additionally, assessing HIF level/activity or its downstream of PHD/HIF axis in the immune cells isolated from these transgenic animals could provide insight into the association of the results with the oxygen sensing pathway, thereby strengthening the findings.**

Response: We agree this would be very interesting to include in future work and thank the reviewer for this suggestion.

**It is unclear why the authors mainly utilized the LPS+normoxia/hypoxia model (but no infection model at all) in studying the role of PHD1, whereas the LPS+normoxia model (without hypoxia condition) was only briefly used prior to shifting focus to primarily investigate infection models for PHD3 study.**

Response: We agree it will be of interest to investigate the role of neutrophil PHD1 expression in infection responses and hope to carry out this work in the future but elected to make available our current data rather than delay to include future work on infection models. We have prioritised the PHD3 work due to the interesting phenotypes we have observed and were keen to publish this in a timely fashion in order that the data are available to the wider hypoxia research community.

**Several inconsistencies in experimental designs across different studies are noticed without providing adequate reasons. For example, although metabolic profiles were obtained in both studies, the Seahorse experiments were exclusively conducted in PHD3 studies, while energy status measurement (ATP/ADP level) was only conducted in the PHD1 study. **  

Response: We have not yet carried out seahorse assays on the PHD1 deficient neutrophil for the same reasons described above. These assays require a high number of cells to be used and therefore high mouse numbers which we did feel able to justify given the subtle changes noted in the data presented here.

**Moreover, certain assays such as MPO or NETosis were not performed in the PHD1 *ex vivo* study, whereas chemotaxis was not observed in the PHD3 *ex vivo* study. Additionally, while neutrophil degranulation (e.g., MPO and elastase concentration) was evaluated mainly in *in vivo* studies, it was not assessed in *ex vivo* studies at all. The BAL albumin was used to assess vascular leakage in the lung for sterile inflammation in Fig. 3, but the BAL IgM was obtained to evaluate vascular leakage in the lung for bacterial infection in Fig. 5.** **Throughout the manuscripts, the authors neglected to include control groups in most experiments to assess whether the deficiency of the gene impacted baseline parameters. For example, it remains unclear whether PHD1 ^fl/fl^MRP8Cre ^+/-^ mice exhibited neutrophil numbers at the baseline comparable to PHD1 ^fl/fl^MRP8Cre ^-/-^ control animals in Fig. 1, or whether the absence of PHD1 in neutrophils altered the oxygen consumption rate or any metabolic state of neutrophils at the baseline (without LPS treatment) in Fig. 2?** 

Response: We thank the reviewer for highlighting these limitations and apologise for any confusion due to the use of different assays between experiments. It is important to note that in healthy, naïve mice, there are no neutrophils found in the BAL ^8-10^ and therefore it is impossible to compare baseline tissue neutrophils in this system. The isolation of neutrophils from the blood of mice is possible but the numbers are small and so it is challenging to carry out e.g. LC-MS metabolic studies on these cell populations whilst operating within the 3Rs principles. Bone marrow neutrophils are more numerous but the degree to which they are representative of mature transmigrated neutrophils is at best uncertain and therefore we did not feel that detailed phenotyping of bone marrow neutrophils would provide additional relevant data. We have not carried out *ex-vivo* degranulation assays as, 24 hours following LPS induced lung injury (or indeed 14 hours post-pneumonia induction), the neutrophils will have already undergone their maximal degranulation and so we instead measure the granule proteins in the BAL supernatant as a surrogate of neutrophil degranulation.

**Figure 1 is somewhat ambiguous. The sole difference observed between PHD1 ^fl/fl^MRP8Cre ^+/- ^vs PHD1 ^fl/fl^MRP8Cre ^-/-^ was a slight increase in neutrophils recruitment in PHD1 ^fl/fl^MRP8Cre ^+/- ^mice after LPS-induced lung injury in hypoxia. However, this conclusion was drawn without knowledge of the basal lung neutrophil levels of these two strains. The title of Fig. 1L, specified as the “resolution” of neutrophils numbers, was confusing as it was uncertain if the value indeed reflected the resolution (PHD1 ^fl/fl^MRP8Cre ^+/- ^mice appear to have better resolution, yet the value displayed was lower). The issue could be raised from the finding that the levels of BAL neutrophils in PHD1 ^fl/fl^MRP8Cre ^-/-^ mice remained unchanged between 24 hours and 48 hours (Fig. 1I and 1K), with no identified basal level of lung neutrophil number. This could be attributed to the possibility that the neutrophils were not recruited to the lungs after all the challenges in these PHD1 ^fl/fl^MRP8Cre ^-/-^ mice, thus obviating the need for resolution.** 

Response: As noted above, at baseline there are no BAL neutrophils and so we don’t think that baseline numbers are an explanation for the differences seen. In figure 1N in the revised manuscript we have tried to demonstrate that the rate at which neutrophils are lost from the airways (which we term resolution of neutrophil numbers) is higher and therefore the ratio of the 48 hour counts to the 24 hour counts is lower, the value which is shown in the graph. We apologise that this was not clear. In response to another reviewer’s comments, we have now also included the resolution data for the normoxic animals as well and hope this will improve clarity (Figure 1O in the revised manuscript). The mean BAL neutrophil numbers for the PHD1 ^fl/fl^MRP8Cre ^-/-^ in hypoxia are 2.63 million at 24 hours and 3.21 million at 48 hours. In contrast, in the PHD1 ^fl/fl^MRP8Cre ^-/+^ animals the mean values were 3.37 million at 24 hours and 2.66 million at 48 hours. Hypoxia is a well-known survival stimulus for neutrophils and the persistence of neutrophils in the airways of hypoxic mice is consistent with this and with our previously published data.

**Additionally, concerning Fig. 1, it is unclear why the authors opted to segregate the graphs displaying the results of normoxia (Fig. 1E-1G, Fig. 1M-1N) vs hypoxia (Fig. 1H-1J, Fig. 1O-1P) at 24 hours while merging both normoxia and hypoxia conditions for the 48-hours studies (Fig. 1K and Fig. 1Q). The concerns were raised considering the different statistical analysis used for 24 hours (Mann-Whitney test) vs 48 hours (ordinary one-way ANOVA).** 

Response: We apologise for this confusion regarding the presentation of the data. When the 48 hour data is analysed by Mann-Whitney (as per the 24 hour data), it remains non-significant. Given that no significant differences were observed, we have compressed the 48 hour data into single graphs to make the data more reader friendly.

**Although the authors illustrated that the expression of PHD3, but not PHD1 and PHD2, was significantly upregulated in neutrophils isolated from animals maintained in hypoxia (Figure 1C, 3A), none of the experiments in this manuscript explore the role of PHD3 in hypoxic conditions. Despite investigating the effects of PHD1 deficiency in neutrophils under both normoxic and hypoxic conditions, it is unexpected that all subsequent experiments regarding PHD3 functions were conducted using PHD3 ^fl/fl^LysMCre ^+/-^ mice solely in normoxia.**

Response: We have previously published on PHD3 deficiency in hypoxic neutrophils ^5^ and further characterisation of the impact of PHD3 loss on hypoxic conditions is of course of great interest but beyond the scope of this article. Given the existing literature regarding PHD1 deficiency which consistently demonstrates a protective effect in hypoxic or ischaemic injury, we felt it was important to assess the role of PHD1 in both normoxic and hypoxic neutrophils and to report our overall negative findings in this cell type.

**Regarding the pneumococcal infection model depicted in Fig.5, clarification is needed regarding why the authors examined colony-forming units (CFU) only 14 hours after infection, a time point that may be too early to observe differences in terms of neutrophil function/recruitment (again, no baseline level of neutrophil number is provided, thus it is difficult to assess recruitment). At this early stage, it is worth considering whether the difference in CFU could potentially be attributed to the depletion of PHD3 in the other resident myeloid cells such as alveolar macrophages?**

Response: Extensive and detailed characterisation has been performed on pneumococcal challenge models and we have based our experimental protocol on the standard recognised in this field ^11,12^. There is significant mortality by day 2 following inoculation in the high dose pneumococcal model (>50% ^12^) which is the reason for culling the mice at the 14 hour time point. There is already significant neutrophil recruitment to the airways in this model by 14 hours (as shown in Figure 5D). It is of course important to consider the impact of other myeloid cells, including alveolar macrophages, in this model. Although the literature would suggest that their role is in the resolution of pneumonia rather than the acute phase with depletion of the alveolar macrophage pool resulting in increased mortality 72 hours after infection but no difference in lung or blood CFU counts at 20 hours post-infection ^13^. In future work it will of course be important to repeat the *in vivo* infection models in the neutrophil specific lines, in order to confirm that PHD3 loss specifically in the recruited neutrophils is responsible for the observed phenotypes. We have now addressed this in the discussion and limitations section.

**A number of aspects of the Methods section and experimental design require careful attention. Detailed protocols for several parts are notably absent. **

- **Most *in vitro* studies in the manuscript relied on either isolated bone marrow neutrophils or isolated BAL neutrophils. However, the Methods section lacks comprehensive information on how to isolate and purify these cells. While it was mentioned that “Bone marrow neutrophils were isolated by percoll gradient followed by fluorescence activated cell sorting to ensure sufficient purity.” in the legend of Fig. 1, specific details such as how to perform percoll gradient, whether BAL samples underwent percoll gradient enrichment, or the FACS sorting process, including the markers used to label neutrophils, were not provided in the Methods section. The authors should also mention if the purity of the neutrophils post-purification was confirmed.**

Response: We apologise that these details were not in the original manuscript and have updated the methods section to provide more comprehensive information about neutrophil isolation protocols. We have included it below for convenience:   Bone marrow: Both hind limbs were dissected out and stored in a clean bijou container on ice until processed. Each bone was flushed using 10ml of 1xHBSS with 0.2% BSA. The cell suspension was resuspended, passed through a 70mm filter into a fresh 50ml polystyrene falcon tube and then centrifuged at 450G for 10 minutes. 90% vol/vol percoll stock was made using 10X HBSS with NaHCO _3_ and this stock was used to make 81%, 62% and 55% percoll with 1x HBSS. All three percoll preparations were layered in a 15ml falcon tube and the cells were resuspended in 3ml of 1xHBSS with 0.2% BSA which was layered on top of the 52% percoll layer. The gradient was centrifuged at 2000G for 30 minutes (acceleration 1, deceleration 0). Bone marrow monocyte precursors were removed from the top layer with a pastette. Neutrophils were removed from the middle layer and washed in 30ml of 1xHBSS with 0.2%BSA. Hypotonic saline red blood cell lysis was carried out. Following the percoll purification above, neutrophil purity was found to be approximately 90%. In order to generate highly pure bone marrow neutrophils samples, cells from the neutrophil layer were resuspended in 1xHBSS with 0.2% BSA and purified by fluorescence-activated cell sorting (FACS) based on the forward/side scatter profile and auto-fluorescence as previously described ^14^ Bronchoalveolar lavage: Where highly pure BAL neutrophils were required, we used a previously optimized discontinuous percoll gradient coupled with a red cell lysis step in order to gain purities of >98%.  BAL cells were first pelleted by centrifugation at 350G for 10 minutes. BAL samples from 1-3 mice (depending on the final cell number required) were pooled onto a single percoll gradient. 90% vol/vol percoll was made using 10X PBS and further percoll solutions were made using this stock 90% percoll mixed with 1X PBS. 3ml of 78% percoll was pipetted into the bottom of a 15ml falcon tube, followed by 3ml of 69% percoll. The cell pellet(s) were resuspended in 3ml of 52% percoll which was then layered onto the 69% layer. The gradient(s) were centrifuged at 1200G for 30mins with an acceleration of 1 and deceleration of 0. Following centrifugation, the macrophages accumulated on the top of the 52% layer and were removed using a sterile pastette. The neutrophils (within the 69% layer) were then removed using a fresh sterile pastette and placed into a 50ml polystyrene falcon tube. This was topped up to 50ml with sterile PBS to wash off the percoll and the purified neutrophils pelleted at 350G for 8 mins. Residual red cells were lysed by hypotonic saline lysis. A cytospin was made prior to the final centrifugation step to assess purity by morphology. During optimisation of this protocol, purity was also confirmed by flow cytometry and the neutrophils were found to be >98% pure.    

- **In the “LPS lung injury” section: details regarding the conditions/treatments used as control, such as nebulizing the animals with PBS, or the method of inducing normoxia compared to hypoxia, are lacking. Also, in the statement: “Mice were treated with lipopolysaccharide (LPS) from *Pseudomonas aeruginosa* 10”, not sure what “10” means?**

Response: We did not utilise a PBS nebulisation control as this does not result in neutrophil recruitment to the airways and so does not provide useful control data. The normoxic mice were simply housed in standard conditions, breathing room air via a standard individually ventilated cage. In contrast, as per the methods, the hypoxic mice were housed in a hypoxic cabinet with an inspired oxygen concentration of 10%. The LPS is sourced from *Pseudomonas aeruginosa* (10) ( https://www.sigmaaldrich.com/GB/en/product/sigma/l9143). We apologise that the omission of the brackets may have made this unclear and have updated the manuscript for clarity.    

- **The Methods in “Phagocytosis” section lack details of how flow cytometry was employed to assess phagocytosis and obtain %phagocytosis (in Fig. 2K, 6B, 6C). The term "ice control" shown in the X-axis of Fig. 2K requires explanation. Including representative flow cytometry analysis figures of phagocytosis could enhance clarity. Moreover, while the method section specifies the use of “fluorescently labelled, heat-killed *E. Coli* (Invitrogen)” for the study which I believe shown in Fig. 2K, it did not mention the Staphylococcus and Streptococcus strains used in the phagocytosis assay in Fig. 6B and 6C. These should be mentioned in the Methods section as well.**

Response: We apologise for the lack of clarity here. The ice control is a standard control in this assay to distinguish non-specific binding of the fluorescent bacteria to the outside of the cell from true uptake by phagocytosis. In this data it does not provide useful experimental data and so, to avoid confusion and for consistency with other phagocytosis data in the manuscript we have replotted the data without the ice controls. The species used for the PHD3 phagocytosis assay were D39 *S. pneumonia *and Wood strain without protein A *S. aureus*. We apologise that these were not included in the methods and have now added them to the relevant section.  

- **For NETosis assay, more details are needed on how Sytox Red could be used to assess NET formation and NETosis by flow cytometry. It is unclear what percentage of NETosis means. What was the control of this NETosis study? Were isolated neutrophils challenged with any bacteria/insults to stimulate the release of NETs before fixing the cells with PFA?**

Response: SyTox red is a cell impermeable dye which binds to nucleic acids. We agree that it is not specific for NETosis but is an accepted surrogate marker for this. Percentage NETosis is the percentage of cells which were positive for red fluorescence. It is true that this will also include some cells which have died by necrosis. In order to fully assess NETosis it would be important to use an additional assay such as microscopy or an ELISA to confirm the presence of both DNA and e.g. MPO. Given the lack of difference between genotypes on this initial assay we did not pursue this avenue further. We felt that it was important to include this negative data, accepting the limitations of the assay used. When optimising this assay we trialled both BAL neutrophils with an additional stimulus (PMA) and freshly isolated BAL cells. We found that the addition of PMA did not result in a clearer NETosis signal but did result in really significant cell loss in the assay and so the data presented here are for freshly isolated BAL neutrophils without any further stimulus.  

- **Regarding the respiratory burst assay, the authors used 10 uM fMLP to induce a respiratory burst. However, considering the authors’ previous work [Fig. 2, Sadiku P, et. al. 2017 (Ref 1)], 10uM of fMLP did not activate respiratory burst signal in BAL neutrophils isolated from WT animals. An explanation for this discrepancy between the two studies is warranted. Also, the abbreviation "fMLF" in the Methods section should be changed to "fMLP" for consistency with the abbreviation used in Fig. 2.**

Response: In order to allow the relevant comparison to be made in the referenced paper investigating PHD2, the BAL neutrophils in this context were aged for 6 hours in media prior to being stimulated with fMLF. Whereas in the current study, freshly isolated neutrophils were used. We suggest that this difference in experimental protocol is the cause of the discrepancy in response to fMLF. We apologise for the different abbreviations used and have changed them all to fMLF.  

- **The specification of negative control and positive control for the chemotaxis assay shown in Fig. 2J, along with clarification on how to obtain %positive control, is necessary.**

Response: The negative control is media with no chemoattractant added. The positive control has cell pipetted into the well at the start (instead of onto the membrane). The percentage of positive control is calculated as (cell count in well for experimental condition/cell count in positive control well)*100. Additional details regarding the controls have been added to the methods section.  

- **The authors should provide details on how they analyzed qPCR results to determine relative gene expression. Were they reported as 2^-(ddCT) or just normalized to *Actb* expression? If analyzed using the 2^-(ddCT) method, it is unclear what sample was used as a reference for normalization, as none of the values in Fig. 1A were close to 1. Conversely, if the results were relative to *Actb* expression, it is difficult to believe that PHD3 expression was upregulated to a level 20-fold higher than *Actb* expression. Confirmation of these findings and the methodology used for analysis is necessary.**

Response: The raw data for all qPCR experiments is available on the Figshare associated with this paper. The raw CT values were normalised to b-actin expression. The CT value will of course depend not only on the abundance of the gene of interest but also on the efficiency of the primers and other technical factors, thus we would not try to draw conclusions on the relative abundance of the *Egln *transcripts in comparison to *ActnB *and are using this data only to compare *Egln* transcript expression between treatment groups.  

- **Further elaboration on Seahorse experiments (Fig. 6E-6J) should be included in the Methods section. This includes information on when compounds (glucose, oligo, 2DG) were added to the cells, and at what concentrations, as well as the time point after bacterial infection shown in Fig. 6F and 6I (before or after adding other compounds). Also, did these compound treatments facilitate the explanation of any findings of the story?**

Response: We carried out a modified glycolysis stress test on these cells which involved the addition of bacteria prior to running the standard stress test (Agilent Technologies Kit 103020-100) using the compounds oligomycin and 2-deoxy-glucose at the time points indicated on figures 6E&H. The compound treatments were not relevant to the other findings – they were added to allow calculation of additional factors in the ECAR test but were not significantly different between genotypes. They are not relevant to the OCR measurements but for clarity and transparency have been noted on the figure.    

- **Please verify why the authors used 2-way ANOVA for metabolic profile analysis depicted in Fig. 2A-2F.**

Response: A two way ANOVA allowed us to ascertain whether there was an overall effect of genotype on metabolite abundance and is the test of choice for this type of grouped data with two variables (genotype and metabolite). In response to other reviewer comments, we have additionally carried out post-hoc multiple comparison tests within the ANOVA and found no metabolite reaches significance on its own in these experiments.

**Considering the findings on metabolic activity, it would be beneficial to provide an explanation or discussion on why metabolites in the OxPPP flux were reduced in PHD1 ^fl/fl^MRP8Cre ^+/- ^mice specifically under normoxic conditions (Fig. 2B), while metabolites in TCA cycles were increased in these mice only under hypoxia condition (Fig. 2F).**

Response: Thank you for this suggestion. We have now expanded the discussion section to try to address these findings in more detail.

**Furthermore, in Fig. 6G, there appears to be a trend toward a higher oxygen consumption rate (OCR) in PHD3 ^fl/fl^MRP8Cre ^+/- ^mice even at baseline before bacterial infection. It would be valuable for the authors to address this observation. Additionally, although there was an increase in OCR in these PHD3-depleted neutrophils, no changes were observed in any metabolite intermediates, including those in the OxPPP pathway (Fig. 6M). It would be helpful if the authors could provide an explanation for this discrepancy.**

Response: There is a trend towards higher OCR at baseline in the PHD3fl/fl-MRP8Cre+/- vs PHD3fl/fl-MRP8Cre+/- mice, although this does not reach statistical significance (as shown in Figure 6I). Both genotypes demonstrate an uplift in OCR in response to bacteria. The fold change in OCR is not significantly different between genotypes when calculated as (OCR immediately post-bacteria injection)/(mean OCR pre-bacteria injection) – 1.6 for WT Vs 1.55 for KO. However, when absolute increase in OCR is calculated, i.e. (OCR immediately post-bacteria injection) - (mean OCR pre-bacteria injection), this is significantly different – 267 for WT V 375 for KO, p=0.04, analysed by unpaired t-test. We would suggest that whilst both increase their OCR, the higher level (Figure 6H) and higher absolute increase in the PHD3fl/fl-MRP8Cre+/- (as described above) are indicative of overall increased oxygen consumption in the PHD3 deficient neutrophils which becomes significant with the addition of a bacterial stimulus. We do not see a significant difference in overall ROS production which is in keeping with the unchanged OxPPP metabolites seen here. The production of mROS is not dependent on the oxidative pentose phosphate pathway (as shown in papers which block OxPPP and demonstrate enhanced mROS production ^15^ and so this does not represent a discrepancy.

**Minor concerns: **

- **Please ensure to define all the abbreviations the first time mentioned in the manuscript e.g., IVC, BAL, IT, CFU, MPO, 2DG, all metabolites (e.g., F6P, F16BP, GAO, PEP, etc.)**

Response: Thank you, these have now been added

- **Although the interested gene was intact in these animals, I suggest avoiding to use of the term “wildtype” for all the floxed control animals.**

Response: Thank you, we have removed the term and used the term cre negative and the complete genotype in each case for clarity

- **Methods in “Assessing bacterial load/CFU counts” section: Suggest changing “bijou” to “Bijou container”**

Response: This has been changed.

- **Methods in “Assessment of MPO activity in Staphylococcus aureus skin abscess model” section: Please make sure to italicize *Staphylococcus aureus***- done
- **Methods in “CFSE labelling of *Streptococus pneumoniae*” section: should be “ *Streptococcus*”**-done
- **Please make sure to write the correct format for all mRNA/protein nomenclature with consistency throughout the manuscript. For example, RNA level (mouse) should be written italicized as “ *Egln1*”**-done
- **Figure 1A: please make sure the title is labeled as “PHD2” (looks like PI-D2 in the pdf file), and also label the Y-axis with the gene name in Fig. 1C to be consistent with Fig. 1A, 1B **- done
- **Please change the Y-axis label in Fig.2A, 2D to be similar to Fig. 2B-2C or Fig. 2E-2F**-done
- **Figure 2I-2K needs labels for what black vs gray dots represent. Also, it should be DCF (“I”), chemotaxis (“J”), and phagocytosis (“K”) in the figure legend**-done
- **The authors stated that “ex-vivo BAL cells from the neutrophil specific PHD3fl/flMRP8Cre+/- mouse lines with *Staphylococcus aureus*. We used glucose-depleted media to represent the hypoglycaemic airway”. This is confusing since the authors used *Staphylococcus aureus* for the skin infection model, not the airway infection model.**

Response: The cells used in this assay were *ex-vivo* BAL cells, thus from the airways which are known to be hypoglycaemic. For this reason we assessed phagocytosis in glucose deplete media to recapitulate the environment where the cells originated, rather than to recapitulate the staphylococcal skin model.

- **Figure 4A requires better-quality images – **done
- **Figure 4E needs labels for what black vs white dots represent. What was the time-point after infection of this finding? And what did the p-value mean here?**

Response: The labels have now been added. This data set includes all three time points. The p-value reflects that the slope of the line is “significantly non-zero”.

- **Fig. 4F-4H: why did the authors normalize the CFU per abscess instead of per gram as shown in Fig. 4E? And it is surprising to see that CFU was increased over time from day 2 to day 7. Does this normally occur in this staphylococcal skin infection model?**

Response: The total CFU count was used in order to fully reflect the extent of (or lack thereof) bacterial control in each genotype. Given that the size of the abscess increases over time (as per Figure 4D) it is not surprising that the CFU count per abscess increases over the same time period. These data are in keeping with that of others showing a peak in CFU counts around day 4-6 and enlarging abscesses up to day 5-7 ^16^

- **Fig. 5E: what is “SN” in the title of the figure?**

Response: Thank you for highlighting this, it has been changed to supernatant.

- **Fig. 6A: more N should be added to confirm the findings.**

Response: We accept that this is a small N number. However, each N is generated by pooling the BAL cells from 3-4 mice (per genotype) in order to generate enough cells to run in duplicate and at multiple dilutions for CFU counts. Given that we are able to see a small but significant difference with the data we have generated we didn’t feel it was justifiable within the 3Rs framework to repeat this assay simply to increase numbers.

- **Fig. 6E, 6H: please ensure species names were written in the correct format ( *S. aureus*)**

​​​​​​​ Response: Done.

 References:

 1. Singhal, R. *et al.* Disruption of hypoxia-inducible factor-2α in neutrophils decreases colitis-associated colon cancer. *Am J Physiol Gastrointest Liver Physiol* **326,** G53–G66 (2024).

 2. Patel, R. B. *et al.* Targeting Neutrophil α9 Improves Functional Outcomes After Stroke in Mice With Obesity-Induced Hyperglycemia. *Stroke* **54,** 2409–2419 (2023).

 3. Idol, R. A. *et al.* Neutrophil and Macrophage NADPH Oxidase 2 Differentially Control Responses to Inflammation and to Aspergillus fumigatus in Mice. *J. Immunol.* **209,** 1960–1972 (2022).

 4. Elliott, E. R. *et al.* Deletion of Syk in Neutrophils Prevents Immune Complex Arthritis. *The Journal of Immunology* **187,** 4319–4330 (2011).

 5. Walmsley, S. R. *et al.* Prolyl hydroxylase 3 (PHD3) is essential for hypoxic regulation of neutrophilic inflammation in humans and mice. *J. Clin. Invest.* **121,** 1053–1063 (2011).

 6. Appelhoff, R. J. *et al.* Differential Function of the Prolyl Hydroxylases PHD1, PHD2, and PHD3 in the Regulation of Hypoxia-inducible Factor. *J. Biol. Chem.* **279,** 38458–38465 (2004).

 7. Swain, L. *et al.* Prolyl-4-hydroxylase domain 3 (PHD3) is a critical terminator for cell survival of macrophages under stress conditions. *Journal of Leukocyte Biology* **96,** 365–375 (2014).

 8. Poli, V., Di Gioia, M. & Zanoni, I. Quantitative cytofluorimetric analysis of mouse neutrophil extracellular traps. *STAR Protoc* **4,** 102372 (2023).

 9. Kantrow, S. P., Shen, Z., Jagneaux, T., Zhang, P. & Nelson, S. Neutrophil-mediated lung permeability and host defense proteins. *Am. J. Physiol. Lung Cell Mol. Physiol.* **297,** L738–45 (2009).

 10. Zhao, R., Lopez, B., Schwingshackl, A. & Goldstein, S. A. N. Protection from acute lung injury by a peptide designed to inhibit the voltage-gated proton channel. *iScience* **26,** 105901 (2023).

 11. Dockrell, D. H. *et al.* Alveolar Macrophage Apoptosis Contributes to Pneumococcal Clearance in a Resolving Model of Pulmonary Infection. *The Journal of Immunology* **171,** 5380–5388 (2003).

 12. Marriott, H. M. *et al.* Reactive oxygen species regulate neutrophil recruitment and survival in pneumococcal pneumonia. *Am. J. Respir. Crit. Care Med.* **177,** 887–895 (2008).

 13. Knapp, S. *et al.* Alveolar macrophages have a protective antiinflammatory role during murine pneumococcal pneumonia. *Am. J. Respir. Crit. Care Med.* **167,** 171–179 (2003).

 14. Dorward, D. A. *et al.* Technical advance: autofluorescence-based sorting: rapid and nonperturbing isolation of ultrapure neutrophils to determine cytokine production. *Journal of Leukocyte Biology* **94,** 193–202 (2013).

 15. Daneshmandi, S. *et al.* Blockade of 6-phosphogluconate dehydrogenase generates CD8+ effector T cells with enhanced anti-tumor function. *Cell Rep* **34,** 108831 (2021).

 16. Hruz, P. *et al.* NOD2 contributes to cutaneous defense against Staphylococcus aureus through alpha-toxin-dependent innate immune activation. *Proc. Natl. Acad. Sci. U.S.A.* **106,** 12873–12878 (2009).

# Reviewer report: Reviewer response for version 1

**DOI:** 10.21956/wellcomeopenres.22052.r74192

**Published:** 2024-02-28

**Authors:** Martin Pelletier

**Affiliations:** 1 CHU of Québec Research Center, Quebec, Canada

The manuscript from Watts et al. reports the differential roles of the oxygen-sensing enzymes PHD1 and PHD3 in regulating neutrophil metabolism and function using a combination of in vivo and ex vivo models. On the one hand, the authors report that PHD1 deficiency drives alterations in neutrophil metabolism and recruitment in an oxygen-dependent fashion. Despite this, PHD1 deficiency did not significantly alter *ex vivo* neutrophil phenotypes or *in vivo* outcomes in mouse models of inflammation. On the other hand, PHD3 deficiency was found to enhance neutrophil antibacterial properties without excessive inflammatory responses. This effect was not linked to changes in the abundance of core metabolites but was associated with increased oxygen consumption and increased mitochondrial reactive oxygen species production. The authors conclude that PHD3 deficiency drives a favourable neutrophil phenotype in infection and, as such, is an important potential therapeutic target.

 The work is clearly and accurately presented and cites the current literature. Concerns were raised regarding the study design, the methodological details, the statistical analyses, and the conclusions drawn, as detailed below, and they should be addressed to make the article scientifically sound.

 One of the biggest criticisms is using the two Cre-Lox strategies (LysM- and MRP8-driven Cre-Lox recombination) in the study. Although the authors recognize this as a limitation and justify the use of these two expression models with the fact that they previously utilized both and did not identify a Cre-specific phenotype and therefore determined that using these additional controls was not justified when working within the 3Rs framework, it makes it complex, or almost virtually impossible, to compare the results obtained for PHD1 and PHD3 if the mouse strains, the Cre expression and promoter/enhancer elements of each technology are different. In Fig. 6, mice with both Cre-Lox strategies were used for different experiments and a global conclusion was drawn. In fact, the authors would need to do a set of identical experiments, such as bacterial killing (Fig. 6A) and mROS (Fig. 6O), where significant differences were observed between the PHD3fl/fl-MRP8Cre-/- and PHD3fl/fl-MRP8Cre+/- mice, with both the MRP8 Cre- and LysM Cre-mediated recombinations to prove that the results are the same regardless of the Cre-Lox technology used. In its current format, it is hard to state that the conclusions drawn adequately are supported by the results since the PHD1 and PHD3 experiments were not performed with the same myeloid cell-specific targeted mutants.

 Some methodological details are lacking. The authors state that highly pure neutrophil preparations were used for gene expression and HPLC-MS analysis of metabolites (Seahorse extracellular flux assays also require highly purified preparations, as it is impossible to distinguish the metabolic parameters of different cell populations). The methodology used to obtain these highly pure neutrophil preparations from BALs is not detailed other than using a discontinuous Percoll gradient (mentioned in the HPLC-MS analysis of metabolites section). The same can be said with bone marrow neutrophils used for gene expression (such as Fig. 1A-B), where fluorescence-activated cell sorting was used to ensure sufficient purity. What markers were used, and what purity level qualifies as “sufficient”?  The authors must provide the method and techniques to quantify this high purity and the percentage of purity obtained with their preparations. In addition, the authors should provide details regarding the filter used for the chemotaxis assays (i.e., the size of the pores) as well as how were ATP and ADP levels determined (Fig. 2G-H; data may have been generated by HPLC-MS, but it is not clear and should be detailed in the Mat & Met) or the genotypes of the mice used for Fig. 3A-B.

 Most of the statistical analyses seem adequate. However, the 2-way ANOVA performed in Fig. 2A-F and Fig. 6K-M does not provide detailed information on differences among the various study groups. To fully understand group differences in an ANOVA, the authors should conduct post hoc tests (called multiple comparison analysis) of the differences between particular pairs of experimental and control groups. Moreover, the conclusion of a “non-significant positive association” (page 10) for the result presented in Fig. 5B is inadequate. If no statistical difference is found between the groups then the authors should not conclude that there is a positive association.  

 The sole use of beta-actin as a reference gene is questionable, as one assumes that the expression of this gene is constitutively high and that a given treatment will have little effect on the expression level. The MIQE guidelines (Bustin SA. et al., 2009 [Ref 1]) report that “Normalization against a single reference gene is not acceptable unless the investigators present clear evidence for the reviewers that confirms its invariant expression under the experimental conditions described. The optimal number and choice of reference genes must be experimentally determined, and the method reported”. Indeed, it is often difficult to identify even a single gene that meets this criterion (Glare EM. et al., 2002 [ref 2]; Schmittgen TD et. al., 2000 [Ref 3]; Thellin O. et al., 1999 [Ref 4]). The authors should consider the more accurate strategy for normalization that was proposed by Vandesompele and collaborators (Vandesompele J. et al., 2002 [Ref 5]), and detailed in the MIQE guidelines, to carefully select a set of genes that display minimal variation across the treatment, determine the geometric mean of these genes, and normalize the gene(s) of interest to the geometric mean.

 The strategy used for the sequential injections of drugs in the Seahorse assays should be better explained. What is the rationale for injecting the bacteria, then glucose, oligomycin and 2DG? Why not also report the OCR and ECAR following the injection of glucose? What can be concluded following the injection of oligomycin? Also, the OCR after the bacteria injection is indeed increased in PHD3fl/fl-MRP8Cre+/- vs PHD3fl/fl-MRP8Cre+/- mice (Fig. 6F). However, the OCR before the bacteria injection is also increased in PHD3fl/fl-MRP8Cre+/- vs PHD3fl/fl-MRP8Cre+/- mice (Fig. 6E). To account for this basal difference between the two groups, the authors need to baseline the data before the injection of the bacteria and then report the increase after the injection.

 The authors report that the OCR is increased after bacteria injection in PHD3fl/fl-MRP8Cre+/- vs PHD3fl/fl-MRP8Cre+/- mice (Fig. 6F). After confirming that this uplift in OCR was observed in the context of conserved ECAR (Fig. 6H-J) and equivalent abundance of TCA cycle, glycolysis and OxPPP metabolic intermediaries (Fig. 6K-M; done with the LysM-Cre mice), the authors considered whether this increase in OCR reflected increased ROS production. However, total ROS (Fig. 6N) and mROS production (Fig. 6O), the latter one being increased in PHD3fl/fl-MRP8Cre+/- vs PHD3fl/fl-MRP8Cre+/-, mice were examined in unstimulated cells. How can the authors explain that a similar increase was not observed in baseline OCR (Fig. 6G) in their Seahorse assays? In their discussion, the authors state: “Using Seahorse technology, we identified enhanced oxygen consumption in PHD3 deficient neutrophils upon challenge with bacteria which we have demonstrated is associated with increased mROS production in inflammatory BAL neutrophils.” This is actually an overstatement, as this association was never demonstrated. For this, the whole set of experiments should have been performed with a single genotype (MRP8-Cre mice) and the total and mROS production examined following the activation of cells in the presence of bacteria, as done in the Seahorse assays.

 The authors are most likely aware of the multiple differences between mouse and human neutrophils, and this limitation should be addressed in the discussion, especially when it is concluded that PDH3 represents a potential therapeutic target. Experiments are most likely required with human cells to prove that PDH3 represents, indeed, a potential therapeutic target.

 Corrections are needed regarding the figures and the text:

 1) The manuscript should include only high-resolution figures. For example, the quality of Fig. 1N, 1O and 1P are currently inadequate, as the axis labels are fuzzy.

 2) The figures should clearly mention the experimental group. For example, Fig. 1E and 1H have the same title and axis labels. It is therefore critical to read the figure legend to understand that Fig. 1E refers to normoxia while Fig. 1H refers to hypoxia. The same can be said for Fig. 1F-1I, Fig. 1G-1J, Fig. 1M-1O and Fig. 1N-1P. The authors should combine these results in a single graph, such as in Fig. 1C, 1D or 1Q, or clearly state the experimental group in the figure title.

 3) PMN is used in the axis title of some figures but has not been defined in the text or the figure legend.

 4) The Greek letter Mu should be used instead of the letter “u” for micro.

 5) b-actin should be replaced by beta-actin (using the Greek letter) in the legend of Fig. 1.

 6) FMLP should be replaced by fMLF in Fig. 2I, as this is the abbreviation used in the text.

 7) In the legend of Fig. 5, the text “BAL supernatant from PHD3fl/flLysMCre+/- and PHD3fl/flLysMCre-/- mice subject to IT…” should be corrected to “BAL supernatant from PHD3fl/flLysMCre+/- and PHD3fl/flLysMCre-/- mice subject **ed** to IT…”.

 8) IT should be defined in the text.

 9) In Fig. 6E-G, the axis label states OCR (mpH/min). In Seahorse assays, OCR is actually provided as nmoles/min. The axis label needs to be corrected.

Is the work clearly and accurately presented and does it cite the current literature?

Yes

If applicable, is the statistical analysis and its interpretation appropriate?

Partly

Are all the source data underlying the results available to ensure full reproducibility?

Yes

Is the study design appropriate and is the work technically sound?

Partly

Are the conclusions drawn adequately supported by the results?

Partly

Are sufficient details of methods and analysis provided to allow replication by others?

Partly

Reviewer Expertise:

Neutrophils, immunometabolism, inflammation

I confirm that I have read this submission and believe that I have an appropriate level of expertise to confirm that it is of an acceptable scientific standard, however I have significant reservations, as outlined above.

## References

[1] : The MIQE guidelines: minimum information for publication of quantitative real-time PCR experiments. *Clin Chem* .2009;55(4) : 10.1373/clinchem.2008.112797 611-22 10.1373/clinchem.2008.112797 19246619

[2] : beta-Actin and GAPDH housekeeping gene expression in asthmatic airways is variable and not suitable for normalising mRNA levels. *Thorax* .2002;57(9) : 10.1136/thorax.57.9.765 765-70 10.1136/thorax.57.9.765 12200519 PMC1746418

[3] : Effect of experimental treatment on housekeeping gene expression: validation by real-time, quantitative RT-PCR. *J Biochem Biophys Methods* .2000;46(1-2) : 10.1016/s0165-022x(00)00129-9 69-81 10.1016/s0165-022x(00)00129-9 11086195

[4] : Housekeeping genes as internal standards: use and limits. *J Biotechnol* .1999;75(2-3) : 10.1016/s0168-1656(99)00163-7 291-5 10.1016/s0168-1656(99)00163-7 10617337

[5] : Accurate normalization of real-time quantitative RT-PCR data by geometric averaging of multiple internal control genes. *Genome Biol* .2002;3(7) : 10.1186/gb-2002-3-7-research0034 RESEARCH0034 10.1186/gb-2002-3-7-research0034 12184808 PMC126239

# Response

**Published:** 2024-08-05

**Authors:** Emily Watts

**Affiliations:** Institute for Regeneration and Repair, The University of Edinburgh, Edinburgh, Scotland, UK

We would like to thank the reviewer for their time and their detailed review of this manuscript. We have submitted these data to Wellcome Open Research as we wanted to share both the interesting positive findings, but also the important negative data, with the scientific community in a timely fashion. We agree that some of the findings are preliminary and that we have not fully dissected the mechanisms underlying the phenotypes which we have observed. Nevertheless, we felt that the phenotype observed in the PHD3 deficient mice was of sufficient interest to publish the data in its current form. Further studies will of course be required to confirm the mechanisms underpinning these findings. We are providing some limited new experimental data in this revised version but the majority of the comments raised have been addressed in the attached rebuttal and by revision of the text and conclusions drawn. We are grateful to the reviewer for their recognition of clarity and accuracy of our work and hope that in this revised version they will agree that the conclusions drawn are supported by the data.    

**The manuscript from Watts et al. reports the differential roles of the oxygen-sensing enzymes PHD1 and PHD3 in regulating neutrophil metabolism and function using a combination of in vivo and ex vivo models. On the one hand, the authors report that PHD1 deficiency drives alterations in neutrophil metabolism and recruitment in an oxygen-dependent fashion. Despite this, PHD1 deficiency did not significantly alter *ex vivo* neutrophil phenotypes or *in vivo* outcomes in mouse models of inflammation. On the other hand, PHD3 deficiency was found to enhance neutrophil antibacterial properties without excessive inflammatory responses. This effect was not linked to changes in the abundance of core metabolites but was associated with increased oxygen consumption and increased mitochondrial reactive oxygen species production. The authors conclude that PHD3 deficiency drives a favourable neutrophil phenotype in infection and, as such, is an important potential therapeutic target.**

**The work is clearly and accurately presented and cites the current literature. Concerns were raised regarding the study design, the methodological details, the statistical analyses, and the conclusions drawn, as detailed below, and they should be addressed to make the article scientifically sound.**

**One of the biggest criticisms is using the two Cre-Lox strategies (LysM- and MRP8-driven Cre-Lox recombination) in the study. Although the authors recognize this as a limitation and justify the use of these two expression models with the fact that they previously utilized both and did not identify a Cre-specific phenotype and therefore determined that using these additional controls was not justified when working within the 3Rs framework, it makes it complex, or almost virtually impossible, to compare the results obtained for PHD1 and PHD3 if the mouse strains, the Cre expression and promoter/enhancer elements of each technology are different. In Fig. 6, mice with both Cre-Lox strategies were used for different experiments and a global conclusion was drawn. In fact, the authors would need to do a set of identical experiments, such as bacterial killing (Fig. 6A) and mROS (Fig. 6O), where significant differences were observed between the PHD3fl/fl-MRP8Cre-/- and PHD3fl/fl-MRP8Cre+/- mice, with both the MRP8 Cre- and LysM Cre-mediated recombinations to prove that the results are the same regardless of the Cre-Lox technology used. In its current format, it is hard to state that the conclusions drawn adequately are supported by the results since the PHD1 and PHD3 experiments were not performed with the same myeloid cell-specific targeted mutants.**

Response: We agree that there are challenges in comparing the data between these different Cre lines due to their different specificities but felt that the PHD1 data was important negative data which we wished to make available to other researchers working in the field of hypoxia. This work was carried out separately from the PHD3 work and our aim in this paper was not necessarily to directly compare PHD1 with PHD3 deficiency, rather to present the data from both genotypes for interest and completeness. The data from the LysMCre line in figure 6 demonstrate no difference in phagocytosis between genotypes. We have reworded the conclusions and limitations sections in order to improve clarity and to better explain the use of the different mouse lines.

**Some methodological details are lacking. The authors state that highly pure neutrophil preparations were used for gene expression and HPLC-MS analysis of metabolites (Seahorse extracellular flux assays also require highly purified preparations, as it is impossible to distinguish the metabolic parameters of different cell populations). The methodology used to obtain these highly pure neutrophil preparations from BALs is not detailed other than using a discontinuous Percoll gradient (mentioned in the HPLC-MS analysis of metabolites section). The same can be said with bone marrow neutrophils used for gene expression (such as Fig. 1A-B), where fluorescence-activated cell sorting was used to ensure sufficient purity. What markers were used, and what purity level qualifies as “sufficient”?  The authors must provide the method and techniques to quantify this high purity and the percentage of purity obtained with their preparations.**

Response: We apologise for this omission. These details have now been included in the manuscript and, for convenience are also detailed here: Bone marrow: Both hind limbs were dissected out and stored in a clean bijou container on ice until processed. Each bone was flushed using 10ml of 1xHBSS with 0.2% BSA. The cell suspension was resuspended, passed through a 70mm filter into a fresh 50ml polystyrene falcon tube and then centrifuged at 450G for 10 minutes. 90% vol/vol percoll stock was made using 10X HBSS with NaHCO _3_ and this stock was used to make 81%, 62% and 55% percoll with 1x HBSS. All three percoll preparations were layered in a 15ml falcon tube and the cells were resuspended in 3ml of 1xHBSS with 0.2% BSA which was layered on top of the 52% percoll layer. The gradient was centrifuged at 2000G for 30 minutes (acceleration 1, deceleration 0). Bone marrow monocyte precursors were removed from the top layer with a pastette. Neutrophils were removed from the middle layer and washed in 30ml of 1xHBSS with 0.2% BSA. Hypotonic saline red blood cell lysis was carried out. Following the percoll purification above, neutrophil purity was found to be approximately 90%. In order to generate highly pure bone marrow neutrophils samples, cells from the neutrophil layer were resuspended in 1xHBSS with 0.2% BSA and purified by fluorescence-activated cell sorting (FACS) based on the forward/side scatter profile and auto-fluorescence as previously described ^1^. Bronchoalveolar lavage: Where highly pure BAL neutrophils were required, we used a previously optimized discontinuous percoll gradient coupled with a red cell lysis step in order to gain purities of >98%.  BAL cells were first pelleted by centrifugation at 350G for 10 minutes. BAL samples from 1-3 mice (depending on the final cell number required) were pooled onto a single percoll gradient. 90% vol/vol percoll was made using 10X PBS and further percoll solutions were made using this stock 90% percoll mixed with 1X PBS. 3ml of 78% percoll was pipetted into the bottom of a 15ml falcon tube, followed by 3ml of 69% percoll. The cell pellet(s) were resuspended in 3ml of 52% percoll which was then layered onto the 69% layer. The gradient(s) were centrifuged at 1200G for 30mins with an acceleration of 1 and deceleration of 0. Following centrifugation, the macrophages accumulated on the top of the 52% layer and were removed using a sterile pastette. The neutrophils (within the 69% layer) were then removed using a fresh sterile pastette and placed into a 50ml polystyrene falcon tube. This was topped up to 50ml with sterile PBS to wash off the percoll and the purified neutrophils pelleted at 350G for 8 mins. Residual red cells were lysed by hypotonic saline lysis. A cytospin was made prior to the final centrifugation step to assess purity by morphology. During optimisation of this protocol, purity was also confirmed by flow cytometry and the neutrophils were found to be >98% pure.

**In addition, the authors should provide details regarding the filter used for the chemotaxis assays (i.e., the size of the pores) as well as how were ATP and ADP levels determined (Fig. 2G-H; data may have been generated by HPLC-MS, but it is not clear and should be detailed in the Mat & Met) or the genotypes of the mice used for Fig. 3A-B.**

Response: We apologise for these omissions in methodological details. The pore size was 5mm and this detail has been added. The ATP and ADP levels were indeed measured as part of the HPLC-MS experiments and this has now been clarified.

**Most of the statistical analyses seem adequate. However, the 2-way ANOVA performed in Fig. 2A-F and Fig. 6K-M does not provide detailed information on differences among the various study groups. To fully understand group differences in an ANOVA, the authors should conduct post hoc tests (called multiple comparison analysis) of the differences between particular pairs of experimental and control groups. **  

Response: We have performed these post-hoc, multiple comparison analyses and found that, despite there being a significant difference overall between the genotypes in the metabolic pathways detailed, no individual metabolite is significantly different between genotypes. The manuscript has been updated to include this additional information.  

**Moreover, the conclusion of a “non-significant positive association” (page 10) for the result presented in Fig. 5B is inadequate. If no statistical difference is found between the groups then the authors should not conclude that there is a positive association.** Response: Thank you for this comment, we apologise for this and this conclusion has been removed.

**The sole use of beta-actin as a reference gene is questionable, as one assumes that the expression of this gene is constitutively high and that a given treatment will have little effect on the expression level. The MIQE guidelines (Bustin SA. et al., 2009 [Ref 1]) report that “Normalization against a single reference gene is not acceptable unless the investigators present clear evidence for the reviewers that confirms its invariant expression under the experimental conditions described. The optimal number and choice of reference genes must be experimentally determined, and the method reported”. Indeed, it is often difficult to identify even a single gene that meets this criterion (Glare EM. et al., 2002 [ref 2]; Schmittgen TD et. al., 2000 [Ref 3]; Thellin O. et al., 1999 [Ref 4]). The authors should consider the more accurate strategy for normalization that was proposed by Vandesompele and collaborators (Vandesompele J. et al., 2002 [Ref 5]), and detailed in the MIQE guidelines, to carefully select a set of genes that display minimal variation across the treatment, determine the geometric mean of these genes, and normalize the gene(s) of interest to the geometric mean.** Response: Thank you for this helpful information regarding the qPCR data which we will certainly take into consideration in future work. In order to increase confidence that the data we present here is valid we have reviewed our own and others’ proteomic datasets (immpres.co.uk) and found no significant variation in b-actin expression in different neutrophil populations in these data sets. Additionally, Zhang and colleagues ^2^ and Ledderose and colleagues ^3^ have also investigated this important question in neutrophil populations and concluded that b-actin is a suitable reference gene in neutrophils.

**The strategy used for the sequential injections of drugs in the Seahorse assays should be better explained. What is the rationale for injecting the bacteria, then glucose, oligomycin and 2DG? Why not also report the OCR and ECAR following the injection of glucose? What can be concluded following the injection of oligomycin? ** 

Response: We carried out a modified glycolysis stress test on these cells which involved the addition of bacteria prior to running the standard stress test (Agilent Technologies Kit 103020-100) using the compounds oligomycin and 2-deoxy-glucose at the time points indicated on figures 6E&H. The compound treatments were not relevant to the findings regarding oxygen consumption which are detailed in Figure 6 – they were added to allow calculation of additional factors in the ECAR. The addition of glucose allows calculation of baseline glycolysis, oligomycin allows calculation of glycolytic capacity and finally, the addition of 2-DG allows glycolytic reserve to be calculated. None of these parameters were different between genotypes or with the addition of bacteria and so have not been plotted in this paper. They are not relevant to the OCR measurements of interest but for clarity and transparency have been noted on the figure. 

**Also, the OCR after the bacteria injection is indeed increased in PHD3fl/fl-MRP8Cre+/- vs PHD3fl/fl-MRP8Cre+/- mice (Fig. 6F). However, the OCR before the bacteria injection is also increased in PHD3fl/fl-MRP8Cre+/- vs PHD3fl/fl-MRP8Cre+/- mice (Fig. 6E). To account for this basal difference between the two groups, the authors need to baseline the data before the injection of the bacteria and then report the increase after the injection.**

Response: There is a trend towards higher OCR at baseline in the PHD3fl/fl-MRP8Cre+/- vs PHD3fl/fl-MRP8Cre+/- mice, although this does not reach statistical significance (as shown in Figure 6I). Both genotypes demonstrate an uplift in OCR in response to bacteria. The fold change in OCR is not significantly different between genotypes when calculated as (OCR immediately post-bacteria injection)/(mean OCR pre-bacteria injection) – 1.6 for WT Vs 1.55 for KO. However, when absolute increase in OCR is calculated, i.e. (OCR immediately post-bacteria injection) - (mean OCR pre-bacteria injection), this is significantly different – 267 for WT V 375 for KO, p=0.04, analysed by unpaired t-test. We would suggest that whilst both increase their OCR, the higher level (Figure 6H) and higher absolute increase in the PHD3fl/fl-MRP8Cre+/- (as described above) are indicative of overall increased oxygen consumption in the PHD3 deficient neutrophils.

**The authors report that the OCR is increased after bacteria injection in PHD3fl/fl-MRP8Cre+/- vs PHD3fl/fl-MRP8Cre+/- mice (Fig. 6F). After confirming that this uplift in OCR was observed in the context of conserved ECAR (Fig. 6H-J) and equivalent abundance of TCA cycle, glycolysis and OxPPP metabolic intermediaries (Fig. 6K-M; done with the LysM-Cre mice), the authors considered whether this increase in OCR reflected increased ROS production. However, total ROS (Fig. 6N) and mROS production (Fig. 6O), the latter one being increased in PHD3fl/fl-MRP8Cre+/- vs PHD3fl/fl-MRP8Cre+/-, mice were examined in unstimulated cells. How can the authors explain that a similar increase was not observed in baseline OCR (Fig. 6G) in their Seahorse assays?**  

Response: This is an interesting observation and we thank the reviewer for highlighting this potential discrepancy. As noted above, although the trend in higher OCR is present at baseline, it is only when neutrophils are further stimulated with bacteria that the difference becomes significant. We would hypothesise that the total OCR will comprise not only mROS but also cytosolic ROS production, fatty acid oxidation and other oxygen requiring metabolic processes. The small but significant increase in mROS production at baseline may therefore be masked by these other oxygen consuming processes which may not be different between genotypes. It would be very interesting to further test this hypothesis by repeating the mROS experiment with the addition of bacteria and this will be addressed in future work. In addition, although the neutrophils in the *ex-vivo *ROS and mROS studies were not stimulated with bacteria, they were harvested from the lungs following an LPS induced lung injury and so were activated rather than unstimulated cells.  

**In their discussion, the authors state: “Using Seahorse technology, we identified enhanced oxygen consumption in PHD3 deficient neutrophils upon challenge with bacteria which we have demonstrated is associated with increased mROS production in inflammatory BAL neutrophils.” This is actually an overstatement, as this association was never demonstrated. For this, the whole set of experiments should have been performed with a single genotype (MRP8-Cre mice) and the total and mROS production examined following the activation of cells in the presence of bacteria, as done in the Seahorse assays.**  

Response: Thank you for this feedback, we have now rewritten these sections of the discussion and expanded the limitations sections to avoid overstating the associations which we have observed.

**The authors are most likely aware of the multiple differences between mouse and human neutrophils, and this limitation should be addressed in the discussion, especially when it is concluded that PDH3 represents a potential therapeutic target. Experiments are most likely required with human cells to prove that PDH3 represents, indeed, a potential therapeutic target.**  

Response: Thank you for this helpful suggestion, we have now included these caveats in the limitations section of the paper.

****

**Corrections are needed regarding the figures and the text:**

**1) The manuscript should include only high-resolution figures. For example, the quality of Fig. 1N, 1O and 1P are currently inadequate, as the axis labels are fuzzy.**  

Response: This issue has now been corrected and we apologise for the variable quality of the images.

**2) The figures should clearly mention the experimental group. For example, Fig. 1E and 1H have the same title and axis labels. It is therefore critical to read the figure legend to understand that Fig. 1E refers to normoxia while Fig. 1H refers to hypoxia. The same can be said for Fig. 1F-1I, Fig. 1G-1J, Fig. 1M-1O and Fig. 1N-1P. The authors should combine these results in a single graph, such as in Fig. 1C, 1D or 1Q, or clearly state the experimental group in the figure title.**  

Response: Thank you for this feedback, we have now clearly labelled Fig 1E-F as relating to normoxic data and Fig 1H-J as relating to hypoxic data and hope this will make the data easier to view.

**3) PMN is used in the axis title of some figures but has not been defined in the text or the figure legend.**  

Response:  Thank you, PMN has been replaced with neutrophil in all figures.

**4) The Greek letter Mu should be used instead of the letter “u” for micro.**  

Response: Thank you, this has been corrected.

**5) b-actin should be replaced by beta-actin (using the Greek letter) in the legend of Fig. 1.**

Response: Thank you, this has been corrected.

**6) FMLP should be replaced by fMLF in Fig. 2I, as this is the abbreviation used in the text.**

Response: Thank you, this has been corrected.

**7) In the legend of Fig. 5, the text “BAL supernatant from PHD3fl/flLysMCre+/- and PHD3fl/flLysMCre-/- mice subject to IT…” should be corrected to “BAL supernatant from PHD3fl/flLysMCre+/- and PHD3fl/flLysMCre-/- mice subjected to IT…”.**

Response: Thank you, this has been corrected.

**8) IT should be defined in the text.**

Response: Thank you, this has been corrected in the methods section.

**9) In Fig. 6E-G, the axis label states OCR (mpH/min). In Seahorse assays, OCR is actually provided as nmoles/min. The axis label needs to be corrected.**

Response: We apologise for this error and thank the reviewer for noting it. This has been corrected to pmol/min to best display our data.

 References

 1. Dorward, D. A. *et al.* Technical advance: autofluorescence-based sorting: rapid and nonperturbing isolation of ultrapure neutrophils to determine cytokine production. *Journal of Leukocyte Biology* **94,** 193–202 (2013).

 2. Zhang, X., Ding, L. & Sandford, A. J. Selection of reference genes for gene expression studies in human neutrophils by real-time PCR. *BMC Mol Biol* **6,** 4–7 (2005).

 3. Ledderose, C., Heyn, J., Limbeck, E. & Kreth, S. Selection of reliable reference genes for quantitative real-time PCR in human T cells and neutrophils. *BMC Res Notes* **4,** 427–11 (2011).
